# Mapping Resilient Landscapes to Climate Change in a Megadiverse Country

**DOI:** 10.1111/gcb.70544

**Published:** 2025-10-10

**Authors:** Milena Fermina Rosenfield, Lucas Jardim, Marina Antongiovanni, Luciano Carramaschi de Alagão Querido, Alisson André Ribeiro, Andrea Sánchez‐Tapia, Priscila Silveira, Levi Carina Terribile, Eduardo M. Venticinque, Ana Luisa Albernaz, Letícia Couto Garcia, Leandro Reverberi Tambosi, Marcos Adami, Fernando Gertum Becker, Maíra Benchimol, Luísa Gigante Carvalheiro, Cintia Cornelius, Geraldo Alves Damasceno‐Junior, Ricardo Dobrovolski, Manuel Eduardo Ferreira, Carlos Roberto Fonseca, José Guilherme Fronza, Angela Terumi Fushita, Adrian Antonio Garda, Heinrich Hasenack, Priscila Lemes, Renata Libonati, Camile Lugarini, Marcia C. M. Marques, Felipe Melo, Alessandro Ribeiro de Morais, Sandra Cristina Müller, Andreza Viana Neri, Rita de Cássia Quitete Portela, Mario Barroso Ramos Neto, Camila Linhares Rezende, Fabio de Oliveira Roque, Thadeu Sobral‐Souza, Mariana M. Vale, Gustavo M. Vasques, Eduardo Vélez‐Martin, Ima Vieira, Fernanda P. Werneck, Edenise Garcia

**Affiliations:** ^1^ The Nature Conservancy Brasil São Paulo Brazil; ^2^ Laboratório de Macroecologia, Instituto de Biociências Universidade Federal Do Jataí Jataí Brazil; ^3^ Departamento de Ecologia Universidade Federal Do Rio Grande Do Norte Natal Brazil; ^4^ Coordenação de Ciências da Terra e Ecologia Museu Paraense Emílio Goeldi Belém Brazil; ^5^ Coordenação de Biodiversidade Instituto Nacional de Pesquisas da Amazônia Manaus Brazil; ^6^ Laboratório de Geoprocessamento Para Aplicações Ambientais, Faculdade de Engenharias, Arquitetura e Urbanismo e Geografia Universidade Federal Do Mato Grosso Do Sul Campo Grande Brazil; ^7^ Centro de Engenharia, Modelagem e Ciências Sociais Aplicadas Universidade Federal Do ABC Santo André Brazil; ^8^ IFZ, Department of Animal Ecology and Systematics Justus‐Liebig Universität Giessen Giessen Germany; ^9^ Laboratório de Ecologia da Intervenção, Instituto de Biociências Universidade Federal Do Mato Grosso Do Sul Campo Grande Brazil; ^10^ Instituto Multidisciplinar Para el Estudio del Medio Ramon Margalef Universidad de Alicante Alicante Spain; ^11^ Divisão de Observação da Terra e Geoinformática (DIOTG), Coordenação Geral de Ciências da Terra (CG‐CT) Instituto Nacional de Pesquisas Espaciais São José dos Campos Brazil; ^12^ Departamento de Ecologia, Instituto de Biociências Universidade Federal Do Rio Grande Do Sul Porto Alegre Brazil; ^13^ Applied Ecology & Conservation Lab, Programa de Pós‐graduação Em Ecologia e Conservação da Biodiversidade Universidade Estadual de Santa Cruz Ilhéus Brazil; ^14^ Departamento de Ecologia, Instituto de Ciências Biológicas Universidade Federal de Goiás Goiânia Brazil; ^15^ Instituto de Ciências Biológicas Universidade Federal Do Amazonas Manaus Brazil; ^16^ Instituto de Biociências Universidade Federal de Mato Grosso Do Sul Campo Grande Brazil; ^17^ Instituto de Biologia Universidade Federal da Bahia Salvador Brazil; ^18^ Laboratório de Processamento de Imagens e Geoprocessamento, Instituto de Estudos Sócio‐Ambientais Universidade Federal de Goiás Goiânia Brazil; ^19^ Natu Capital Florianópolis Brazil; ^20^ Departamento de Botânica e Zoologia Universidade Federal Do Rio Grande Do Norte Natal Brazil; ^21^ Departamento de Botânica e Ecologia, Instituto de Biociências Universidade Federal de Mato Grosso Cuiabá Brazil; ^22^ Departamento de Meteorologia, Instituto de Geociências Universidade Federal Do Rio de Janeiro Rio de Janeiro Brazil; ^23^ Núcleo de Gestão Integrada Instituto Chico Mendes de Conservação da Biodiversidade Antonina Brazil; ^24^ Laboratório de Ecologia Vegetal Universidade Federal Do Paraná Curitiba Brazil; ^25^ Universidade Federal de Pernambuco Recife Brazil; ^26^ School of Animal, Rural and Environmental Sciences Nottingham Trent University Southwell UK; ^27^ Laboratório de Ecologia, Evolução e Sistemática de Vertebrados Instituto Federal Goiano Rio Verde Brazil; ^28^ Laboratory of Ecology and Evolution of Plants, Department of Plant Biology, Programa de Pós‐Graduação Em Botânica Universidade Federal de Viçosa Viçosa Brazil; ^29^ Departamento de Ecologia, Instituto de Biologia Universidade Federal Do Rio de Janeiro Rio de Janeiro Brazil; ^30^ UR Forêts et Sociétés Centre de Coopération Internationale en Recherche Agronomique Pour le Développement Montpellier France; ^31^ Embrapa Solos Rio de Janeiro Brazil; ^32^ Ilex Consultoria Científica Porto Alegre Brazil; ^33^ Coordenação de Botânica Museu Paraense Emilio Goeldi Belém Brazil

**Keywords:** biodiversity, climate adaptation, climate resilience, connectivity, conservation, geodiversity, restoration

## Abstract

The effects of global climate change on biodiversity and ecosystem functioning are unevenly distributed in the geographic space. Identifying sites more suitable to sustain biodiversity in a changing climate is essential to both species conservation and restoration strategies at different scales. Here, we map terrestrial climate‐resilient sites for biodiversity across Brazil to identify sites with greater chances of providing suitable conditions for species to persist under regional climate change. Our mapping combines spatial metrics based on landscape heterogeneity, a proxy for microclimatic variability, and local connectedness, a measure of connectivity between habitats, to determine landscape resilience, assuming that resilience to climate change will be greater the more heterogeneous the characteristics of local habitats are and the more connected they are in the landscape. Our results show that within each biome, medium to high resilient sites are mostly found in the Amazon (40% of the biome) and Pantanal (38%). Low resilience, conversely, is concentrated in the Atlantic Forest (41% of the biome), followed by Cerrado (37%), Pampa (36%), and Caatinga (34%). Landscape resilience information has the potential to be used to effectively guide decision‐making and public policy on strategies for conservation, restoration, and sustainable use practices. Priority for conservation should be on high resilience sites as they have the potential to sustain biodiversity in face of undergoing and future climate change. Other approaches could be used in situations of medium to low resilience also, such as: conservation of current corridors in sites with high local connectedness, but low landscape heterogeneity; restoration of natural vegetation on sites that show high landscape heterogeneity, but low local connectedness; and sustainable practices in areas of low resilience. Our study provides an updated method to pinpoint climate‐resilient sites for biodiversity which was applied to a megadiverse country but is applicable to any ecosystem around the globe.

## Graphical abstract

Our mapping of terrestrial climate‐resilient sites for biodiversity across Brazil identifies sites with greater chances of providing suitable conditions for species to persist under regional climate change. Landscape resilience information has the potential to be used to effectively guide decision‐making and public policy on strategies for conservation, restoration and sustainable use practices at different spatial scales. Our study provides an updated method to pinpoint climate‐resilient sites for biodiversity which was applied to a megadiverse country but is applicable to any ecosystem around the globe.

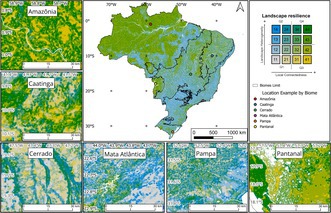

## Abstract (pt)

Os efeitos das alterações climáticas globais na biodiversidade e no funcionamento dos ecossistemas estão distribuídos de forma desigual no espaço geográfico. A identificação de locais mais adequados para sustentar a biodiversidade num clima em mudança é essencial tanto para a conservação das espécies quanto para estratégias de restauração em diferentes escalas. No presente estudo, nós mapeamos ambientes terrestres resilientes ao clima com o foco na biodiversidade em todo o Brasil, para identificar locais com maiores chances de fornecer condições adequadas para que espécies persistam sob mudanças climáticas regionais. Nosso mapeamento combina métricas espaciais baseadas na heterogeneidade da paisagem, um indicador da variabilidade microclimática, e na conectividade local, uma medida da conectividade entre habitats, para determinar a resiliência da paisagem, assumindo que a resiliência às mudanças climáticas será maior quanto mais heterogêneas forem as características dos habitats locais e quanto mais conectados eles estiverem na paisagem. Nossos resultados mostram que, dentro de cada bioma, os locais com resiliência média a alta são encontrados principalmente na Amazônia (40% do bioma) e no Pantanal (38%). Já a baixa resiliência está concentrada na Mata Atlântica (41% do bioma), seguida pelo Cerrado (37%), Pampa (36%) e Caatinga (34%). A informação sobre a resiliência da paisagem tem potencial para ser utilizada para orientar de forma eficaz a tomada de decisões e as políticas públicas sobre estratégias de conservação, restauração e práticas de uso sustentável. A prioridade para conservação deve ser dada aos sítios de alta resiliência, uma vez que têm potencial para manter a biodiversidade face às alterações climáticas atuais e futuras. Outras abordagens podem também ser utilizadas em situações de resiliência média a baixa, tais como: conservação dos corredores atuais em locais com alta conectividade local, mas baixa heterogeneidade da paisagem; restauração da vegetação natural em locais que apresentam alta heterogeneidade da paisagem, mas baixa conectividade local; e práticas sustentáveis em locais de baixa resiliência. Nosso estudo fornece um método atualizado para identificar áreas resilientes a mudanças climáticas para a biodiversidade, que foi aplicado a um país megadiverso, mas que é relevante a qualquer ecossistema ao redor do mundo.

## 1 Introduction

Climate change has altered global and regional temperature, and rainfall regimes, consequently impacting the phenology, behavior, and geographic distribution of species (Antão et al. 2022; Hannah 2021; Vitasse et al. 2018). The main driver of current climate change are greenhouse gas emissions caused by excessive burning of fossil fuels but also by anthropogenic land use‐cover changes (Curtis et al. 2018; IPCC 2022; Lapola et al. 2023). These changes also affect natural ecosystems, causing significant environmental degradation, alteration in habitat quantity and quality, and disrupt regional climate. Shifts in species' geographic distribution are among the main impacts caused by climate change (Abreu‐Jardim et al. 2021; Lemes et al. 2014; Ramalho et al. 2023), which modify communities' composition, disrupting both interspecific interactions (Fricke et al. 2022; Gilman et al. 2010; Memmott et al. 2007), and, consequently, the distribution of functional attributes (Bender et al. 2019). Beyond the impact of climate change, habitat loss and fragmentation are among the main threats to the erosion of biological diversity and, ultimately, cause species extinctions (Díaz et al. 2019; Harfoot et al. 2021; Pardini et al. 2018), both by reducing carrying capacity and by decreasing landscape connectivity, affecting metapopulation and metacommunity dynamics, and community integrity (Banks‐Leite et al. 2014; Chase et al. 2020; Haddad et al. 2015; Hanski and Ovaskainen 2000). This, in turn, compromises the provision, regulation and support of ecosystem functions, structure, and dynamics (Montoya and Raffaelli 2010). However, the consequences of climate change are not uniform across the globe, with ecosystems and species likely exhibiting differences in their resiliences depending on different factors (Manes et al. 2021).

Studies on the impact of climate change on biodiversity usually rely on models that combine species occurrence and climate data to project species distributions at different time periods and climatic scenarios. These studies use global and regional models to project future impacts, enabling the identification of the most vulnerable sites or areas most resistant to climate fluctuations, such as climate refugia (Terribile et al. 2012). However, most climate models provide data at global and regional scales, and rarely downscale to finer resolutions (Fick and Hijmans 2017; Karger et al. 2017; Lima‐Ribeiro et al. 2015), limiting the diagnosis and development of mitigation strategies at local scales, where actions must be effectively developed. A complementary approach to climate models is the use of topography, geodiversity, and physiographic aspects (Anderson et al. 2014; Lawler et al. 2015; Theobald et al. 2015) to diagnose, map, and develop solutions to mitigate the impact of climate change. Species' vulnerability to environmental changes depends on their level of exposure to environmental and biotic variables, their sensitivity, and the capacity to adapt to such changes, through genetic‐level adaptive potential, niche flexibility, or dispersal capacity (Azevedo et al. 2024; Foden et al. 2019; Williams et al. 2008). According to the degree of exposure and a set of biological conditions, species can remain where they are or seek out more suitable areas, depending on the speed with which the climate changes locally and regionally and their ability to disperse (Loarie et al. 2009).

Landscape geodiversity, characterized by the set of abiotic variables of the Earth's surface and subsoil (e.g., topography, geology, pedology, and hydrology; Gray 2004), tends to have a low rate of change in the timeframe feasible for climate change mitigation actions (Dobrowski 2011; Lawler et al. 2015; but see Gill et al. 2015). These variables are part of the factors determining local microclimates, such as the surface that receives the most solar radiation, the position on the relief most exposed to winds, the direction of cold winds, the accumulation of moisture, and water retention in the soil (Dobrowski 2011). The local interaction between the atmosphere and topography results in topoclimates, which can buffer changes from regional climate. Sites where the topoclimate allows microclimate characteristics to be maintained, regardless of regional changes, have been defined as microclimatic refuges (Ashcroft et al. 2012; Dobrowski 2011). In addition, sites with greater topographic complexity and elevation range provide greater variability of environmental conditions for species (Lawler et al. 2015; Rahbek et al. 2019; Tukiainen et al. 2023), and have the potential to support greater biodiversity (Antonelli et al. 2018; Fine 2015; Rahbek et al. 2019; Tukiainen et al. 2019; Vernham et al. 2023). These microclimatic refuges are also highly relevant for people's adaptation and mobility in the context of socio‐ecological systems, particularly for traditional and indigenous communities coping with extreme changes in environmental variables [e.g., extreme drought and heat waves (IPCC 2022; Kakinuma et al. 2019; Murphy 2015)]. In other words, the potential that topoclimates have to act as a microclimatic refuge can buffer changes in the regional climate, making these sites more resilient to climate change. Consequently, understanding the impacts of climate change locally requires an understanding of its impacts on biodiversity, climate, and geodiversity, as all these variables feed back into each other.

Analyzing landscape resilience (Box 1) offers a spatially explicit perspective that complements broader socio‐ecological resilience frameworks, which often emphasize species or community level responses (Lecina‐Diaz et al. 2024). By focusing on intrinsic environmental attributes—such as microclimatic heterogeneity and structural connectivity—the approach we proposed here highlights the capacity of landscapes to buffer climate impacts, facilitate species persistence, and support adaptive responses over time. It also complements ecosystem resilience approaches by offering a spatial lens that helps identify, at larger scales, the physical and structural conditions that favor the maintenance of biodiversity and the recovery capacity of ecosystems subjected to disturbance (Folke et al. 2004). While not capturing exposure or species‐specific sensitivities directly, landscape resilience can serve as a foundational layer to be integrated with vulnerability and risk assessments (Foden et al. 2019), ultimately supporting more robust, multiscale strategies for adaptation and conservation. Landscape resilience is expected to be higher where there are a variety of microclimate conditions that can be accessed by different species and also locally adapted populations. Landscapes with lower structural connectivity reduce the ability of species to track suitable climatic conditions, increasing the deleterious effects of climatic change, an effect that is stronger for poor dispersers. Besides the biological requirements needed for tracking suitable conditions, species need a spatial network of sites that are not only climatically suitable but also provide low resistance to movement (Borges and Loyola 2020; Mantyka‐Pringle et al. 2012; Tourinho et al. 2022). Moreover, in the context of socio‐ecological systems, traditional and indigenous communities used to move across large, heterogeneous landscapes, utilizing microclimatic refuges for shelter, resource management, and other activities. However, today most of them reside in limited areas due to fragmentation or demarcation of ethnic territories. Hence, adequate conservation strategies and climate change mitigation and adaptation strategies depend therefore on integrative approaches that consider these multiple and synergistic effects of global and regional change on population parameters and species distributions (Díaz et al. 2019; Sirami et al. 2017; Sobral‐Souza et al. 2021).

BOX 1 **Glossary of terms used in this study.**

**Table:** 

Term	Meaning used in this study
Landforms	Topographical features present in the landscape, such as mountain tops, valleys, and gorges. Each topographic feature suffers different levels of exposure to solar radiation, wind, and humidity, and the variability of landforms is used as a proxy for environmental heterogeneity and the diversity of landscape's microclimates.
Landscape	A spatially delimited area with heterogeneity in different aspects (landforms, elevation, resistance, and spatial configuration) capable of influencing the ecological processes within it.
Landscape heterogeneity	An estimate of the variability of habitats and microclimates, defined by landforms, elevation range, wetland score, and richness of soils present in the surroundings of a given location.
Landscape resilience	The capacity of an area to absorb the impacts of climate change on species diversity and ecological functions. A resilient landscape presents variability of conditions that support biodiversity, maintaining fundamental relationships between ecological components, and allows for adaptive change in species composition and ecosystem structure.
Landscape resistance	Difficulty imposed by land use and land cover classes on the movement of organisms.
Local connectedness	A measure of how easy it is for organisms to move between landscape elements (or types of land use and land cover). Local connectedness is influenced both by the resistance of landscape elements to the movement of organisms and by the spatial arrangement of these elements.

Brazil stands out not only for its exceptional biodiversity but also for its vast territorial, climatic, and socio‐ecological diversity, making it a strategic country for investigating landscape resilience to climate change. Home to over 203 million people (IBGE 2023) and six major terrestrial biomes—Amazon, Atlantic Forest, Caatinga, Cerrado, Pampa, and Pantanal—Brazil hosts a wide range of ecosystems, from equatorial rainforests to semiarid shrublands. These regions are subject to differentiated impacts of climate change, such as prolonged droughts in the Amazon (Marengo et al. 2018), rising temperatures in the Cerrado (Lapola et al. 2011), and increase in intensity and frequency of surface air temperature extremes along the western South Atlantic coast (Sanches et al. 2023). Projections indicate that the country could face temperature increases of up to 3°C–6°C, depending on the biome, by the end of the century (MCTI 2013), which could dramatically affect ecosystems, agricultural productivity, and water availability. Additionally, Brazil faces intense anthropogenic pressures, including deforestation, habitat fragmentation, and unsustainable land use practices, which exacerbate biodiversity loss (Barlow et al. 2016; de Lima et al. 2020). These combined challenges reinforce the urgency of identifying resilient areas and developing adaptive strategies tailored to the regional ecological and social context.

Recent theoretical and operational advances, such as the greater availability of accessible information at finer spatial scales (Cavender‐Bares et al. 2020; Zarnetske et al. 2019), allowed geodiversity to emerge as an alternative proxy variable for the development of climate change adaptation strategies (Anderson et al. 2023; Dobrowski 2011; Lawler et al. 2015; Tukiainen et al. 2023). This approach has been classified as “conserving nature's stage”, where geodiversity is relatively stable in time and acts as a background where the biodiversity “actors” and interactions operate (Anderson et al. 2014, 2023; Anderson and Ferree 2010; Lawler et al. 2015). The framework suggests that even though the composition of biological communities will probably change over time, the maintenance of abiotic characteristics that foster and preserve biodiversity would allow biological diversity to be promoted, even if the original components change.

By coupling topographic characteristics and landscape context as proxies for landscape resilience to promote biodiversity conservation, we assessed the degree of resilience to climate change across Brazilian terrestrial ecosystems. We addressed the following questions: how are resilient areas distributed across Brazil and within Biomes? What regions stand out as having high resilient areas, and conversely where are regions that show lower resilience? And where should we focus our attention in terms of conservation strategies across the landscape? In this approach, resilient sites are defined as those with high variability of environmental conditions, that simultaneously have low environmental degradation, great landscape structural connectivity, and therefore would be less impacted by regional climate change, and could be used by species to move across landscapes or as refugia (Anderson et al. 2014, 2023). Specifically, mapping microclimatic heterogeneity and local connectedness among natural patches allows for the identification of areas of greater potential to sustain biodiversity in the future. By including different categories in the final result, that provide clear directions in terms of conservation strategies, our analysis goes beyond a previous mapping exercise carried out in North America (Anderson et al. 2014, 2023), and develops new approaches to refine the method to tropical and subtropical complex and megadiverse regions, such as Brazil.

## 2 Methods

We identified climate resilient sites by combining data on landscape heterogeneity and local connectedness (Rosenfield et al. 2024). We assessed resilience across Brazil, aiming to capture the different regions and types of vegetation grouped in Brazilian biomes (IBGE 2021c): Amazônia (the rainforests in north Brazil), Caatinga (the seasonally dry forests in the northeast), Cerrado (the savannas in central Brazil), Mata Atlântica (the seasonal forests and rainforests along the coast, from south to northeast), Pampa (the grasslands in the south), and Pantanal (the wetlands in the central‐west) (Appendix S1: Figure S1). We only evaluated terrestrial environments. Although rivers, lakes, and wetlands were included in the construction of the landscape heterogeneity and local connectedness layers (as described below), water bodies, and other aquatic ecosystems were masked from the final resilience map. They were, therefore, not classified as any class of landscape resilience. A general overview of the analysis is provided below, and the full description, as well as links to the tutorials for running the analysis, are presented in Appendix S1 of this manuscript.

### 2.1 Landscape Heterogeneity

This study considers landscape heterogeneity as a proxy for the variety of microclimates available in a given location. Landscape heterogeneity summarizes information on: (a) variety of landforms, (b) elevation range, (c) wetland score, and (d) soil richness.

- Landform variety: refers to the variety of topographic features, based on a landform model derived from a digital elevation model (DEM) with a resolution of 90 m (the Merit‐DEM database; see Yamazaki et al. 2017). The classification of landforms is based on four metrics: slope, aspect, topographic position, and moisture accumulation indices, which combine differences in wind speed, solar radiation, sediment deposition, and moisture. The combination of these variables allows the identification of mountain tops, valleys, steep slopes, etc. Landform variety was calculated as the number of landforms within a circular area of 450 m radius around each 90 m cell. This radius size, corresponding to 5 pixels of Merit‐DEM, was iteratively chosen as a scale that maximizes landform differentiation without being too sensitive to variation at finer scales, also guaranteeing sufficient pixels to carry out statistical and spatial analyses.
- Elevation range: represents the range of variation in elevation within a specific region. The elevation range considered in this study corresponds to the residuals from a simple linear regression (between elevation range and landform variety) and represents the component not correlated with landform variety. It is determined from the Merit‐DEM (Yamazaki et al. 2017) by calculating the range (maximum − minimum values) in a circular window of 450 m radius around each 90 m cell.
- Wetland score: for flat areas, where landform variety and elevation range do not allow for the discrimination of microclimatic variation, the density and quantity of wetlands capture topoclimatic variation. We consider that wetlands have an effect in stabilizing microclimates and will contribute to landscape resilience (Anderson et al. 2023; Zhang et al. 2016), even if they are impacted by climate change in the future. We used data from the Global Wetlands Database (Gumbricht et al. 2017), resampled from 231 m to a 90 m resolution. The wetland score is a combination of wetland densities at local and regional scales, and also wetland patchiness at a regional scale, defined as the number of wetlands in the area. These variables capture the distribution of humid areas within the landscape, which are essential for maintaining biological diversity and providing ecological services, such as decomposition, biochemical transformation, storage, and production of living plants and animals (Ryeland et al. 2021; Zedler and Kercher 2005). The wetland score was obtained by averaging the density of wetlands within circular areas of 450 m radius (local scale) and 1170 m radius (regional scale) around each 90 m cell. In sites where wetland patchiness (within a circular area of 1170 m radius) was higher than the previous wetland score, we combined values as the weighted average of local density, regional density, and regional patchiness, weighting local density twice as the other variables.
- Soil richness: in places with low variation in landform variety, elevation range, and wetland score, soil richness becomes an important component of environmental heterogeneity in the landscape that could support biodiversity. Soil richness is the number of dominant and subdominant soil types from the pedology database 1:250.000 of the Brazilian Institute of Geography and Statistics (IBGE 2021b), rasterized to a 90 m resolution image.

The layer of landscape heterogeneity is generated by combining the four variables described above after data standardization (*Z*‐score calculation). Landform variety, elevation range, wetland score, and soil richness were converted to *Z*‐scores, assuming means and standard deviations within moving windows with 200 pixels radius. This ensures the inclusion of distinct physical and environmental conditions, and allows local variations in the composition of the associated biota to be captured, in addition to providing a smooth transition between biomes. This alternative was applied so that it would not be necessary to standardize within the biome and avoid having problems in transitional areas. The combination procedure follows a hierarchical evaluation that considers the additional effect of the following variable in the previous one. The evaluation starts with the *z*‐scores of landform variety; then, for sites with higher elevation range, landscape heterogeneity receives the weighted average of both variables, giving double the weight to landform variety; then the same is done for wetland score and finally for soil richness, comparing every combination of the four variables. For the general calculation of landscape heterogeneity, the weight given to landform variety was higher than any other variable. Only in flat areas, where landform variability is lower, the variable wetland score was added a higher weight in providing landscape variability. However even in these areas with lower landform variety and elevation range we maintained the importance of these variables in the calculation of the landscape heterogeneity by averaging both these variables with the wetland score. More details of this evaluation are provided in Appendix S1 of this manuscript.

### 2.2 Local Connectedness

Local connectedness is a measure of how much resistance landscape elements (or types of land use and land cover) offer to the movement of species. We used the structural differences of the various types of land use and land cover as proxies of resistance, with natural areas being less resistant, and highly impacted areas being more resistant to the movement of terrestrial organisms in the landscape. Connectedness is greater where landscape offers less resistance to the movement of terrestrial organisms, representing the degree of landscape permeability (or the inverse of the degree of resistance) around each cell evaluated. The current approach did not take into account differences in the dispersal capacity of organisms, but relies on the basic assumption that well‐preserved native habitats with similar characteristics in close connection to one another represent better passages and refuges for biota than landscapes where patches of well‐preserved habitat are isolated by matrices of degraded or contrasting habitats and distant from one another.

Based on a land use land cover map of Brazil, we assigned resistance values to each class listed in the database, considering their degree of human intervention and resistance to the movement of organisms. We assigned the lowest values (least resistance) to natural areas and the highest values (greatest resistance) to intensely human‐impacted areas (urban areas), with a gradient of values between the two extremes (Appendix S1: Table S1). We obtained land use land cover data from MapBiomas Project (2023) and complemented it with layers of infrastructure of transport and energy (ANEEL 2023; IBGE 2021a). Depending on their characteristics, rivers can represent barriers to the movement of species across the landscape (Coelho et al. 2022; Hayes and Sewlal 2004), so we assigned different resistance weights to rivers and lakes depending on their width.

Once resistance values had been assigned, we estimated local connectedness as the inverse of the weighted average of the resistances within a circular moving window of 23 pixels (approximately 2070 m), which represents the spatial context in which each pixel is inserted. To calculate the weighted average, we used a linear decay function (kernel filter), which recognizes that closer pixels have a greater influence on each other than more distant pixels, influencing the local connectedness value of each pixel. The kernel filter was applied to the resistance layer and reflects not only the landscape composition but is also influenced by the landscape configuration (i.e., the spatial context) surrounding each pixel. This step was conducted to find paths that offer the least potential resistance to species movement, based on the local context of the landscape.

### 2.3 Landscape Resilience

**FIGURE 1:**
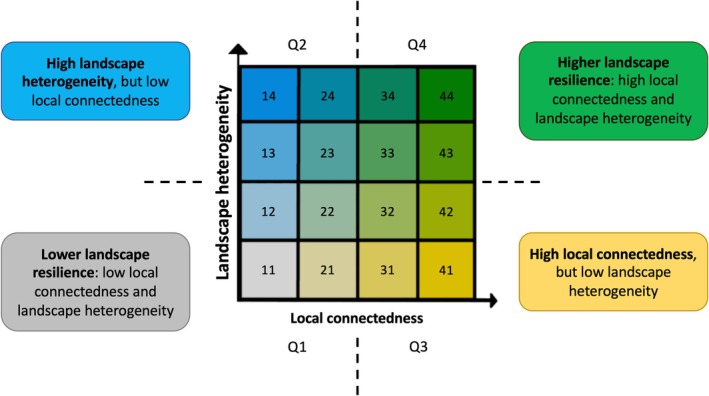
Landscape resilience classification resulting from local connectedness and landscape heterogeneity, showing quadrants based on the combination of metrics quartiles (Q1—gray, Q2—blue, Q3—yellow, and Q4—green) and 16 possible classes (11–44). The classification is based on the distribution of pixels in the original images of landscape heterogeneity and local connectedness.

We calculated landscape resilience by overlaying the maps of landscape heterogeneity and local connectedness based on the distribution of the values of these metrics (classified in quartiles; Appendix S1, Figures S14 and S15). The different classes created by overlaying quartiles on the landscape resilience bivariate scale allow us to identify different areas of interest by the combination of both variables. To facilitate interpretation, the classes generated (11–44) were grouped into four quadrants (Q1‐Q4; Figure 1): quadrant Q1—classes 11, 12, 21, 22; quadrant Q2—classes 13, 14, 23, 24; quadrant Q3—classes 31, 32, 41, 42; and quadrant Q4—classes 33, 34, 43, 44. These quadrants indicate both lower (Q1) and higher (Q4) resilience, and the greater effect of the landscape heterogeneity (Q2) or local connectedness (Q3) layers. From these classes, we generated a bivariate map of landscape resilience, which aims to show the areas where the results of the two metrics converge or diverge, identifying areas where landscape heterogeneity and local connectedness are more important for landscape resilience.

### 2.4 Data Analysis

After generating the data for each layer, we conducted further analysis to explore the results regionally, considering the variations across Brazil and within biomes. For landscape heterogeneity, we assessed the contribution of each component (i.e., landform variety, elevation range, wetland score, and soil richness) to the final value of this layer across Brazil. And for landscape resilience, we evaluated the percentage distribution of quadrants and classes (generated from the distribution of quartiles) across Brazil and within biomes.

## 3 Results

### 3.1 Landscape Heterogeneity

**FIGURE 2:**
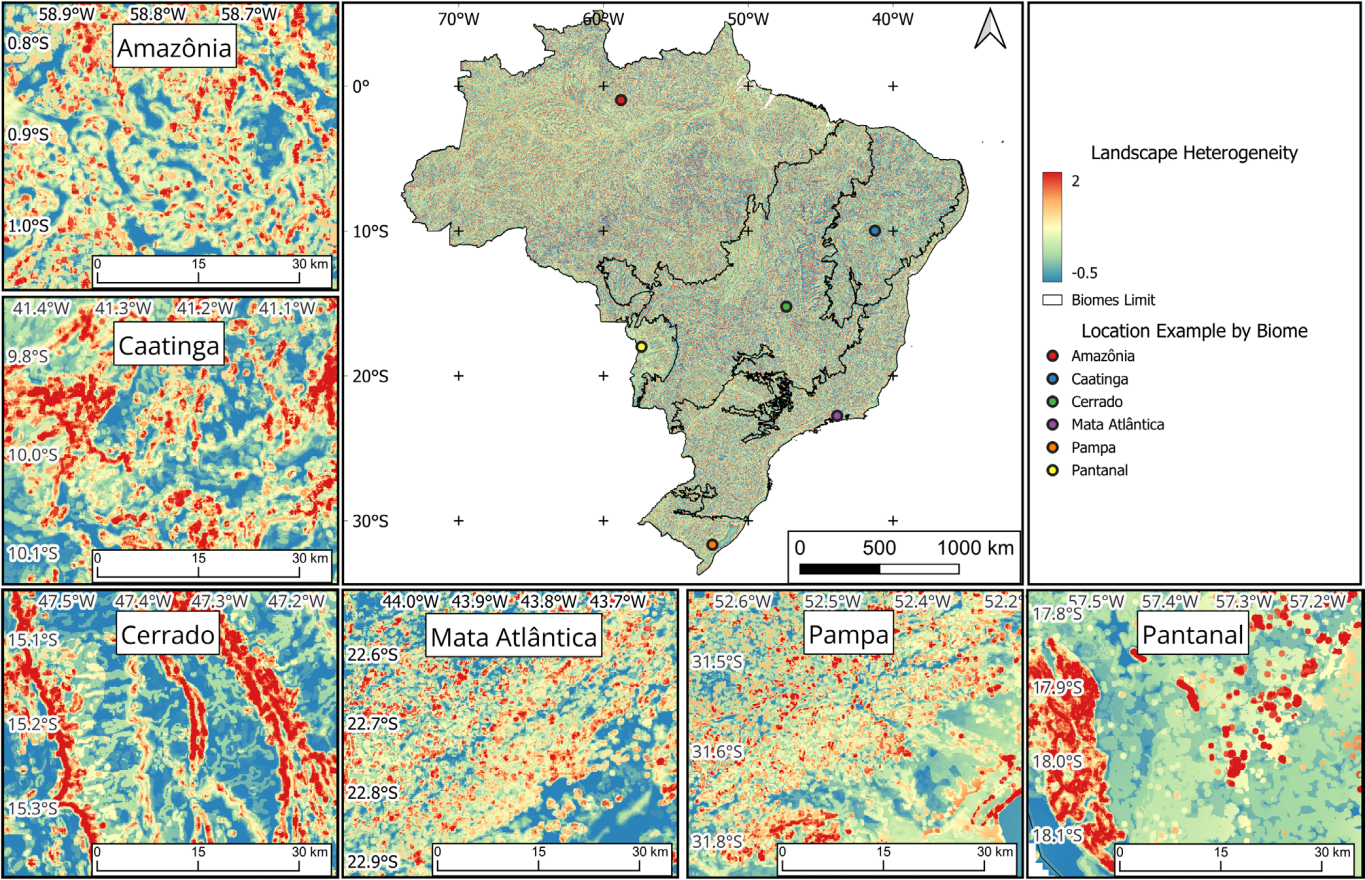
Landscape heterogeneity in Brazil, with a local example for each one of the six biomes, generated from the weighted average of the *Z*‐scores of the variables. Landscape heterogeneity takes into consideration: Landform variety, elevation range, wetland score, and soil richness. Higher and lower values for landscape heterogeneity are shown in a gradient from red to blue. Biome names: Amazônia (Amazon; rainforests), Caatinga (dry forests), Cerrado (savannas), Mata Atlântica (Atlantic forest; seasonal forests and rainforests), Pampa (grasslands), and Pantanal (wetlands). Map lines delineate study areas and do not necessarily depict accepted national boundaries.

We found that areas of high landscape heterogeneity in Brazil are disjointly distributed (Figure 2), highlighting locations with rough terrain, abrupt changes in slope, high density of wetlands, and with different soil types. Due to regional characteristics, the components that make up landscape heterogeneity (i.e., landform variety, elevation range, wetland score, and soil richness) had different contributions depending on the local context. Overall, landform variety was the main variable in determining landscape heterogeneity, followed by the average of all four variables (landform variety, elevation range, wetland score, and soil richness; Appendix S1: Figure S16).

### 3.2 Local Connectedness

**FIGURE 3:**
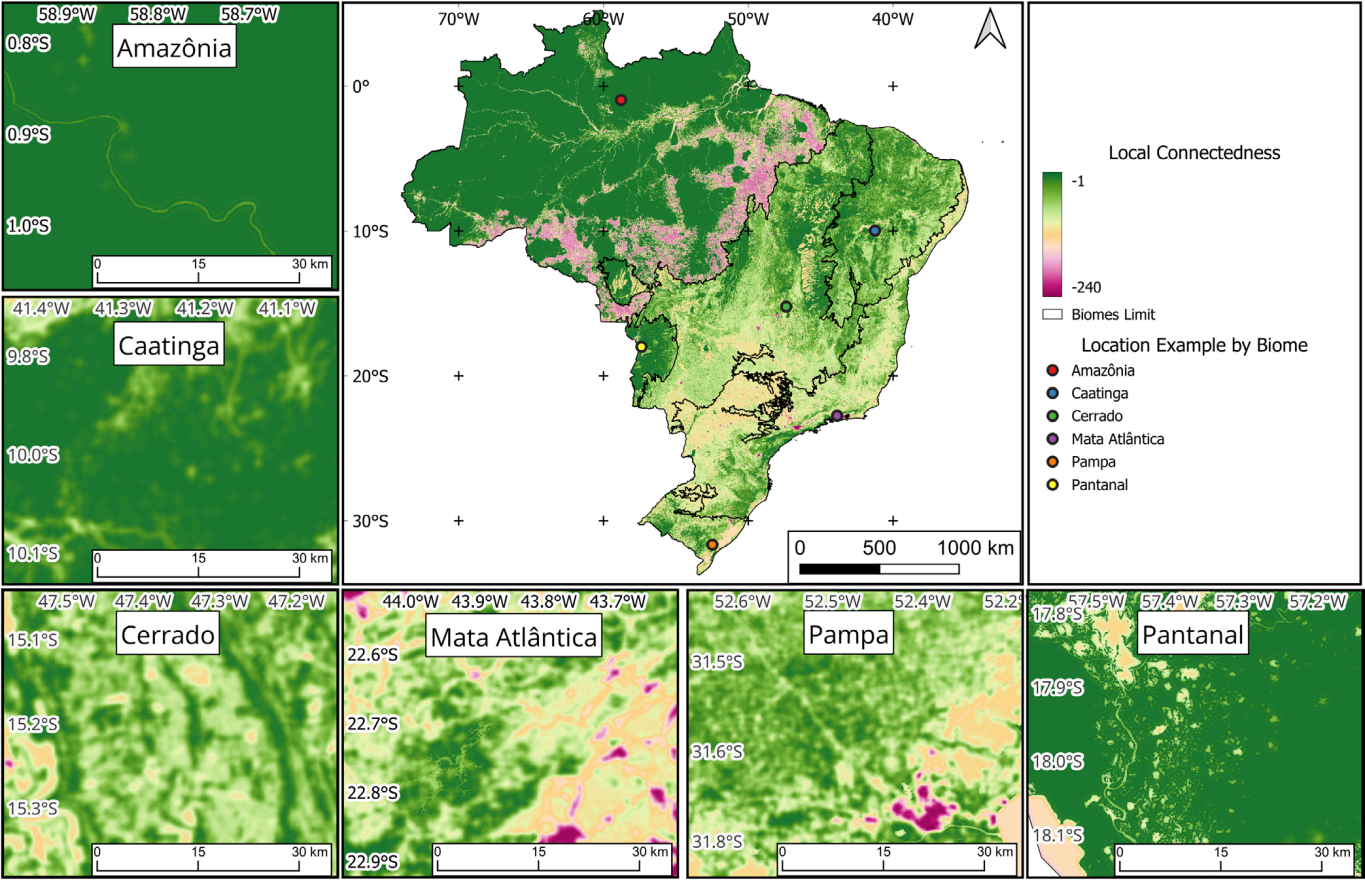
Local connectedness, based on the value of resistance to the movement of organisms conferred by the different types of land use and cover in the context of the landscape. High and low values for local connectedness are shown in green and red, respectively. Biome names: Amazônia (Amazon; rainforests), Caatinga (dry forests), Cerrado (savannas), Mata Atlântica (Atlantic forest; seasonal forests and rainforests), Pampa (grasslands), and Pantanal (wetlands). Map lines delineate study areas and do not necessarily depict accepted national boundaries.

The local connectedness layer has a different pattern to that presented in the landscape heterogeneity layer, showing a division between highly connected regions in the north and others with lower connectedness in the central and southeastern regions of the country (Figure 3). This distribution indicates a clear fragmentation of the Brazilian map into two portions. This is due to the historical occupation of these regions and their current use in terms of agriculture and urban occupation. The pattern of local connectedness reflects the spatial distribution of land use and land cover classes with different resistance values. It is possible to observe a high concentration of classes with high resistance values in the southern and eastern parts of Amazônia (coinciding with the Arc of Deforestation in the Amazon; shown in red in Figure 3), which divides Brazil into two very marked portions. The northwest of the Amazônia biome is the portion with the highest concentration of natural areas, with lower resistance values and therefore high connectedness. The Pantanal also appears as a region with higher local connectedness.

### 3.3 Landscape Resilience

**FIGURE 4:**
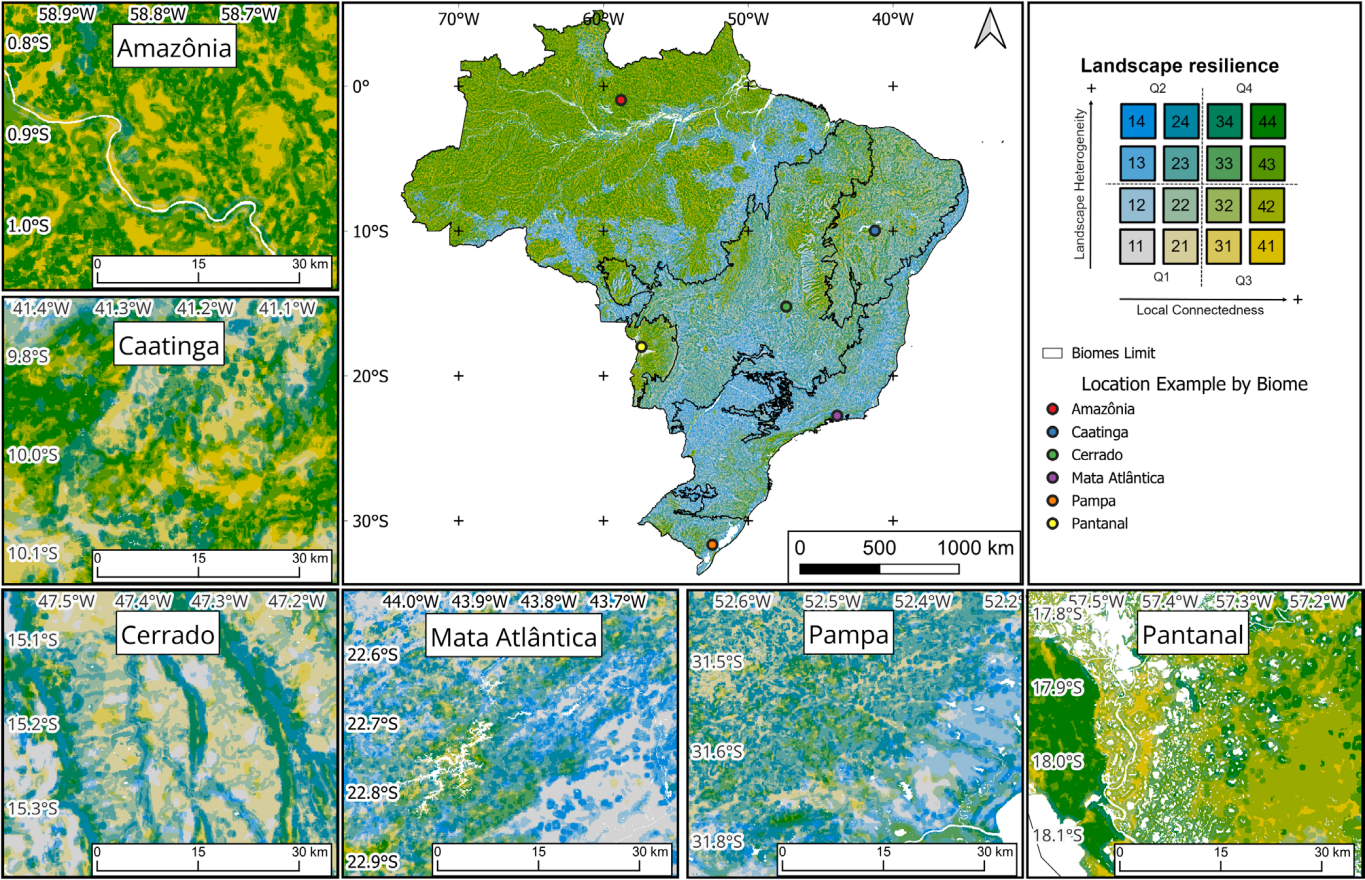
Landscape resilience resulting from the combination of landscape heterogeneity and local connectedness maps, classified by quartiles. The bivariate classification of the landscape resilience layer was divided into four quadrants (Q1, Q2, Q3, and Q4) with different degrees of local connectedness and landscape heterogeneity. White portions in the map, more visible in the biome boxes, represent water bodies. Biome names: Amazônia (Amazon; rainforests), Caatinga (dry forests), Cerrado (savannas), Mata Atlântica (Atlantic forest; seasonal forests and rainforests), Pampa (grasslands), and Pantanal (wetlands). Map lines delineate study areas and do not necessarily depict accepted national boundaries.

Our results provide information on the most suitable areas associated with climate resilience, considering the variability of habitats, and local connectedness between natural vegetation patches. The areas with the highest landscape resilience values are mainly concentrated in Amazônia, in regions close to the Araguaia river basin in the Cerrado biome, in some regions distributed throughout the Pantanal biome, in the interior of the Caatinga, in the southeastern coastal area of Mata Atlântica, and in the regions with the roughest terrain or shallow soils in the Pampa (Figure 4). The spatial pattern of resilience is guided by a more regional pattern of local connectedness, refined by a more local variation in landscape heterogeneity. For example, regions with low anthropogenic impact, such as most regions in Amazônia, vary their resilience levels according to the local distribution of the diversity of the physical environment. Conversely, despite the high heterogeneity of the landscape across Mata Atlântica, its low connectedness reduces the areas of greatest resilience.

The Amazônia biome, due to its size and conservation status, stands out for presenting the highest percentage of areas with both the greatest resilience (quadrant Q4), with 19.5% of the areas assessed in Brazil, and areas with the greatest local connectedness, but low heterogeneity (quadrant Q3), with 17.3% (Table 1). Regarding areas with greater heterogeneity, but low local connectedness (quadrant Q2), the Cerrado and Mata Atlântica stand out, with 7.7% and 6.6% of the total areas assessed in Brazil, respectively (Table 1). As for the areas with the lowest resilience (quadrant Q1), the Cerrado, with 8.8%, is the biome with the highest percentage in Brazil (Table 1). It is important to note that the percentages indicated here refer to the three largest biomes in the country.

**TABLE 1:** Percentages for each quadrant across Brazil and within biomes. Results are relative to the area calculated for Brazil (Across Brazil), and relative to the area of the biome (Within biome).

Quadrant	Amazônia (%)	Caatinga (%)	Cerrado (%)	Mata Atlântica (%)	Pampa (%)	Pantanal (%)
*Across Brazil*						
Quadrant Q1	5.5	3.4	8.8	5.6	0.9	0.2
Quadrant Q2	6.0	2.6	7.7	6.6	0.9	0.1
Quadrant Q3	17.3	2.0	3.3	0.6	0.2	0.7
Quadrant Q4	19.5	2.1	4.0	0.8	0.4	0.7
Total	48.3	10.1	23.8	13.7	2.4	1.8
*Within biome*						
Quadrant Q1	11.4	34.0	37.1	41.1	36.0	13.8
Quadrant Q2	12.3	26.0	32.4	48.4	39.0	7.4
Quadrant Q3	35.9	19.4	14.0	4.3	9.8	40.8
Quadrant Q4	40.4	20.6	16.6	6.2	15.2	38.1
Total	100.0	100.0	100.0	100.0	100.0	100.0

*Note:* Biome names: Amazônia (Amazon; rainforests), Caatinga (dry forests), Cerrado (savannas), Mata Atlântica (Atlantic forest; seasonal forests and rainforests), Pampa (grasslands), and Pantanal (wetlands).

**FIGURE 5:**
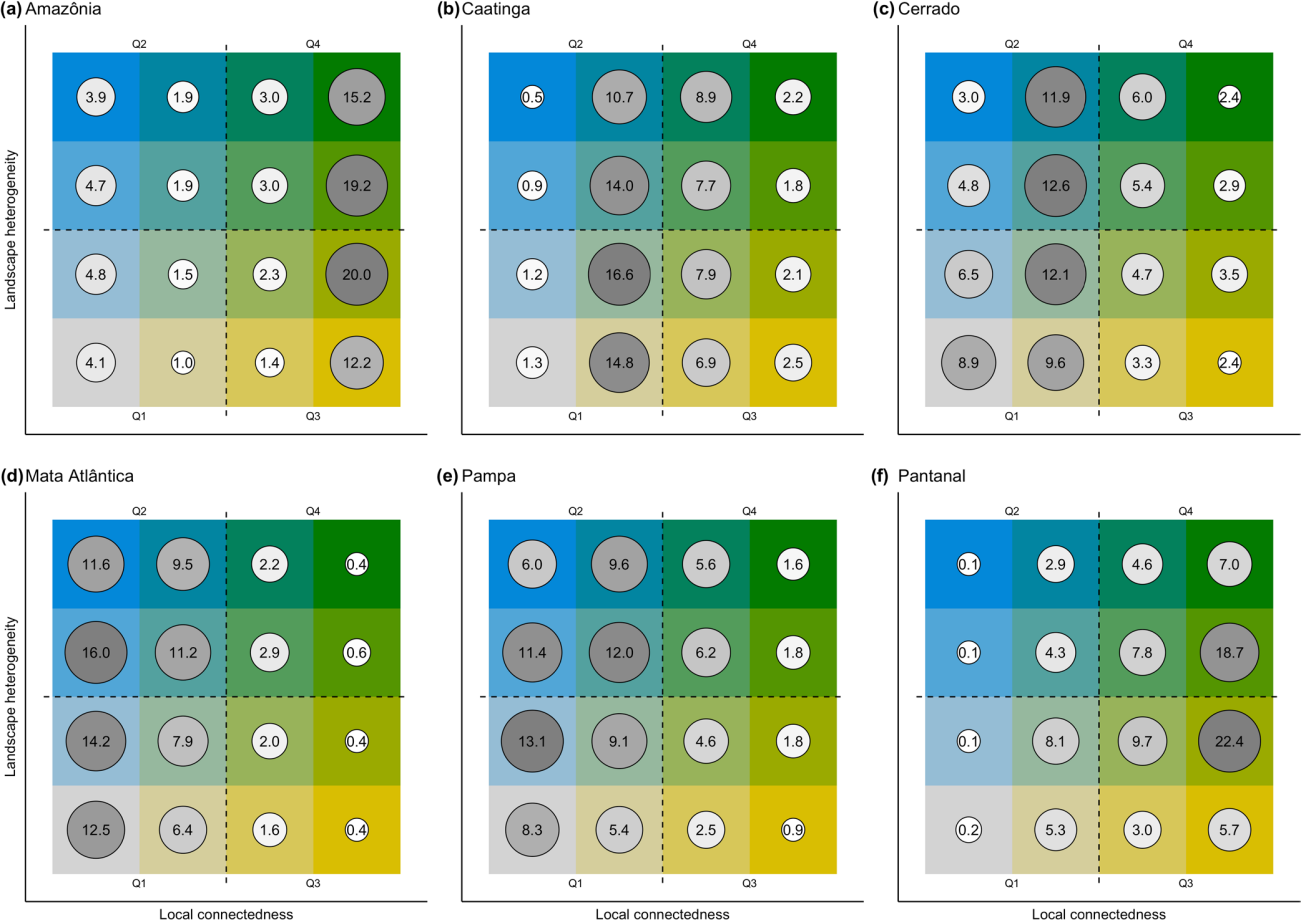
Landscape resilience values by landscape resilience class for each biome: Amazônia (A), Caatinga (B), Cerrado (C), Mata Atlântica (D), Pampa (E), and Pantanal (F). Values shown in circles are the percentage of each class relative to the area of the biome. Larger and darker circles indicate higher percentages. Biome names: Amazônia (Amazon; rainforests), Caatinga (dry forests), Cerrado (savannas), Mata Atlântica (Atlantic forest; seasonal forests and rainforests), Pampa (grasslands), and Pantanal (wetlands).

Considering the context within each biome, and therefore the percentages relative to the area of the biome, Amazônia has the highest percentage of areas with the highest resilience located in quadrant Q4 (40.4%), followed by Pantanal (38.1%), Caatinga (20.6%), Cerrado (16.6%), Pampa (15.2%), and Mata Atlântica (6.2%) (Table 1; Figure 5). Across all biomes, Amazônia has also the majority of the biome in the highest value of local connectedness, distributed along the axis of landscape heterogeneity (classes 41, 42, 43, and 44; Figure 5a). For Caatinga, we find a high concentration of values at intermediate values of the axis of local connectedness, also distributed along the axis of landscape heterogeneity (specially in classes 21–24, but also classes 31–34; Figure 5b). The Cerrado has a similar pattern as the one found in Caatinga, with high concentration in intermediate values of local connectedness (in classes 21–24); however, it is important to highlight that the 5th highest landscape resilience value is found in the class with the lowest landscape resilience (class 11; Figure 5c). For Mata Atlântica, there is a marked pattern of high concentration of values in classes of low local connectedness, distributed along the axis of landscape heterogeneity (in quadrants Q1 and Q2; Figure 5d). The Pampa has a similar pattern to the one found for Mata Atlântica, with high percentages along the classes of low local connectedness (quadrants Q1 and Q2; Figure 5e). And finally, for Pantanal, the pattern is slightly different from all the other biomes, with the highest percentages found in classes with high local connectedness and intermediate landscape heterogeneity (classes 32, 33, 42, and 43; Figure 5f).

In the Amazônia, the areas of greatest landscape resilience are concentrated in the northwest and west portions of the biome. The quadrant with the highest landscape resilience (Q4) represents 40.4% of the pixels assessed in this biome and the very high resilience class (class 44) shows 15.2% of the pixels assessed (Table 1; Figure 5). These values can be considered high when looking at the overall values obtained and represent the highest values when compared to the other biomes in Brazil. In addition, the quadrant with the highest local connectedness (and low landscape heterogeneity; Q3) represents 35.9% of the total, indicating that the conservation pattern of the biome's native vegetation contributes to the high local connectedness and landscape resilience found. This shows the importance of Amazônia for biodiversity conservation in the face of climate change.

In the Caatinga, the areas with the highest landscape resilience (quadrant Q4) represent 20.6% of the total pixels evaluated for the region and are concentrated in the interior portions (Table 1). Unfortunately, only 2.2% of pixels found in this region belong to the very high resilience class (class 44; Figure 5). Areas of low resilience (quadrant Q1), on the other hand, correspond to 34.0% of the pixels assessed (Table 1), indicating places with low landscape heterogeneity and low local connectedness.

In the Cerrado, the areas of greatest landscape resilience are more concentrated in the north and east of the biome, in the northwest, and in the far west. Similarly to the Caatinga region, the quadrant with the highest landscape resilience (Q4) represents 16.6% of the pixels assessed in the region, and the very high resilience class (class 44), only 2.4% (Table 1; Figure 5). Areas of low resilience (quadrant Q1), on the other hand, account for 37.1% of the region (Table 1).

In the Mata Atlântica, a biome with high human population and long‐term history of overexploitation of natural resources, the high resilience classes have the lowest percentage, with only 6.2% of the pixels classified in quadrant Q4 and only 0.4% classified in the very high resilience class (class 44; Table 1; Figure 5). The areas with the greatest landscape resilience are identified in the south and southeast of the region, concentrated in areas of moderate to high elevation and high slope. The quadrant with the highest local connectedness (and low landscape heterogeneity; Q3) also has the lowest value of total pixels assessed in this biome, with only 4.3% (Table 1). On the other hand, the value identified for the quadrant of greatest landscape heterogeneity (and low local connectedness; Q2) stands out, with 48.4% of the pixels evaluated for the biome (Table 1), the highest among all biomes and the highest percentage overall. Therefore, our results show that local connectedness is a determining factor in the resilience pattern currently found for the biome. Finally, the areas of low landscape resilience (quadrant Q1) correspond to 41.1% of the biome's territory (Table 1), highlighting the extent of areas with low local connectedness in areas of low landscape heterogeneity.

In the Pampa, the areas with the highest resilience value are located in the central region, in the west of the biome, and also close to the coastal line. This distribution coincides with the location of the areas with the greatest local connectedness. The quadrant with the highest landscape resilience (Q4) corresponds to 15.2% of the pixels evaluated in the biome and the very high resilience class (class 44), only to 1.6% (Table 1; Figure 5), the second lowest value among all the biomes (behind only to Mata Atlântica, described below). Areas of low resilience (quadrant Q1), in turn, correspond to 36.0% of the pixels evaluated (Table 1). It is important to note the high value for the quadrant with the greatest landscape heterogeneity (and low local connectedness; Q2), 39.0% (Table 1), indicating places with high variability in the landscape but that lack local connectedness.

In the Pantanal, the regions with the highest landscape resilience are mostly those close to the highest density of wetlands, an outstanding feature for this region. This region shows values similar to Amazônia: the highest landscape resilience (quadrant Q1) represents a significant 38.1% of the total pixels evaluated in the region, second only to Amazônia (Table 1). When considering the very high resilience class (class 44), 7% are found in this region (Figure 5). It is important to note that the quadrant with the highest local connectedness (and low landscape heterogeneity; Q3) has a high percentage of 40.8%, indicating places with natural vegetation that allow organisms to move around the landscape.

## 4 Discussion

By applying the conserving nature's stage approach (Beier et al. 2015), we identified sites and regions with the potential of having greater microclimatic variability and connectedness in the landscape, consequently offering microclimatic refuges given their topographical, hydrological, and edaphic characteristics. Since topographic characteristics tend to change little or slowly over time, we believe that areas with greater variation in topographic conditions should be more resilient to predicted climate change. Although the local impact of climate change is defined by the interaction between the atmosphere, biodiversity, and the diversity of the physical environment, the last variable is usually not considered in assessments. Hence, our approach is an essential complement to climatic and ecosystem approaches and provides a new facet for identifying locations that are potentially resilient to climate change, especially at the local level, where microclimate data are commonly scarce.

Our framework for estimating landscape resilience applied to a megadiverse country identifies the most resilient terrestrial landscapes throughout Brazil and within each of its six biomes. Our results show that there are large extensions of resilient landscapes in Brazil, especially in Amazônia, where there is still a high concentration of natural vegetation. Amazônia is the largest biome, and it is where there is the highest concentration of protected areas, representing more than 70% of protected areas in Brazil (Vieira et al. 2019). Protected areas (“Conservation Units” and “Indigenous Lands” in the Brazilian legislation, including quilombolas' territories according to The National Protected Areas Plan) are especially important for conservation by reducing conversion of natural vegetation, and preserving ecosystem services even under climate change conditions (Beattie et al. 2023; Campos et al. 2024; Gonçalves‐Souza et al. 2021). On the other hand, the least resilient areas are concentrated in Mata Atlântica, a biome that has a long history of human impacts and conversion of natural vegetation (Rezende et al. 2018; Vancine et al. 2024). The Mata Atlântica biome presents small aggregates with high resilience values, which are strongly limited by the low local connectedness found in the region. The history of occupation and degradation of Mata Atlântica is potentially one of the main factors responsible for the current low connectedness throughout the territory occupied by this biome, since these are areas that were widely used both for agriculture and for the settlement of the country's first and larger urban centers.

The spatial information generated does not contain spatial subdivisions (e.g., watersheds and political limits). It can be adjusted to support public policies at the national, regional, and even in smaller spatial scales such as states, watersheds, and other spatial units. The flexibility for defining spatial divisions allowed us to identify the differences between biomes that are consequences of varied geological histories leading to higher landscape heterogeneity, but also consequences of human exploitation history, showing important differences in connectedness. Our results also allowed us to identify intrabiome heterogeneity, which is important for delineating specific regional conservation and restoration actions according to the main drivers of landscape resilience in each region.

**FIGURE 6:**
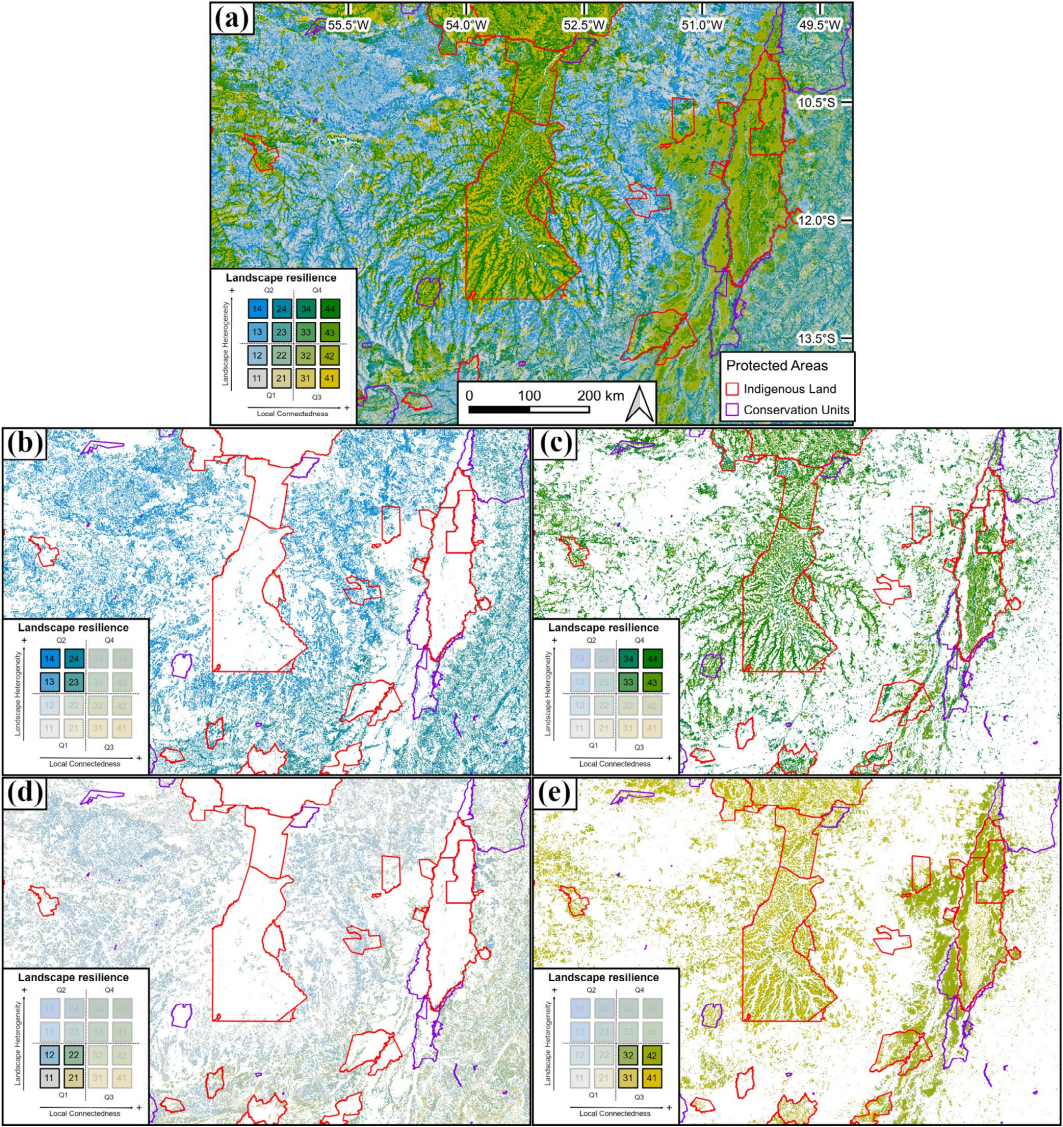
Example of the landscape resilience classification surrounding some protected areas in Brazil (near the Xingu Indigenous Park and the Araguaia National Park, in the state of Mato Grosso). (a) Details highlighting the four quadrants of estimated resilience based on the bivariate color scale are shown in figures (b–e). The order of the maps follows the same representation shown for the quadrants in the landscape resilience diagram: (b) areas classified in quadrant Q2, with high landscape heterogeneity but low local connectedness; (c) the most resilient areas, classified in quadrant Q4, with high landscape heterogeneity and high local connectedness; (d) the least resilient areas, classified in quadrant Q1, with low landscape heterogeneity and low local connectedness; and (e) areas classified in quadrant Q3, with low landscape heterogeneity but high local connectedness.

Different combinations of landscape heterogeneity and local connectedness can lead to landscapes classified at similar intermediate resilience levels but would demand completely different conservation, restoration, and sustainable uses, as well as climate adaptation strategies. Hence, identifying the different combinations of heterogeneity and connectedness is essential to subsidize and delineate effective strategies for specific local and regional landscape conditions. By applying the results of our bivariate resilience classification to multiple objectives, we can break down each quadrant to make it easier to visualize sites within each category and then suggest conservation, restoration, and sustainable use strategies for them. The example presented in Figure 6, for the Xingu Indigenous Park and the Araguaia National Park in the state of Mato Grosso, shows the final resilience map (Figure 6a), as well as the layers separately (Figure 6b–e). In the map, the areas with the greatest resilience (green areas in quadrant Q4; Figure 6c) are those with the greatest potential for conservation in the future, as they indicate places with greater landscape heterogeneity and greater local connectedness, providing better conditions for organisms and species to have more diverse habitats, favoring their persistence in the face of future climate change. These areas have medium to high resilience values (classes 33, 34, and 43) and also very high resilience values (class 44). The latter class represents the highest resilience value among all classes. Sites with high landscape heterogeneity but low local connectedness (blue areas in quadrant Q2; Figure 6b), although not highly resilient, are sites with high potential for increasing resilience, as soon as native vegetation is recovered through restoration actions, a priority for the class 24. Ecological corridors between vegetation remnants and restored areas should be established or preserved to promote the flow of biodiversity. Sites with high local connectedness but low landscape heterogeneity (yellow areas in quadrant Q3; Figure 6e), although not highly resilient, have low resistance to the movement of organisms and more preserved natural vegetation, and are important for current conservation. Moreover, they have the potential to allow the movement and flow of individuals in the landscape and could serve as potential ecological corridors to facilitate adaptation to climate change or displacement to more resilient areas. Finally, sites with low resilience (gray areas in quadrant Q1; Figure 6d), which are those mapped with low landscape heterogeneity and low local connectedness, have low potential for increasing resilience. These sites could be indicated for alternative land uses, ideally sustainable, to provide ecosystem services, generating less impact on biodiversity. However, if there is interest in restoring the ecosystem services provided by these sites, restoration can be considered. In a nutshell, classes that have the highest potential to increase landscape climate change resilience, through restoration actions, are classes 14, 24, and 34, and the highest landscape resilience class, that should be a major priority for conservation, is class 44.

Moreover, the inferred surfaces can be tested under predictive modelling approaches based on organismal data to forecast environmental change impacts on biodiversity (e.g., Azevedo et al. 2024). The use of our data for conservation purposes can be accompanied by other biodiversity data and could be adjusted for each focal species' characteristics (Pressey et al. 2021). For example, species‐specific resistance values can be estimated for different land cover classes to recalculate local connectedness for an endangered species to estimate landscape resilience in its current or future potential distribution. Alternatively, different weights can be used for variables in the landscape heterogeneity index, such as giving higher importance to wetland scores or limiting the elevation range based on the preference of specific organisms to identify priority areas for conservation. While this study focuses only on terrestrial environments, water dynamic components were integrated in the analysis in terms of their influence on the dispersal of terrestrial organisms. For example, in the analysis presented, the different resistance values proposed for bodies of water, considering their width, were based on how difficult it was for terrestrial organisms to move around the landscape.

One additional application of our results is the conservation potential of current protected areas (“Conservation Units”, “Indigenous Lands”, and “Quilombolas' territories”), in addition to other conservation alternatives such as “other effective area‐based conservation measures”—OECMs (Alves‐Pinto et al. 2021), considering the impact of climate change on biodiversity and people. It is possible to identify potential areas for restoration aiming at increasing landscape resilience and promoting the connection between resilient and conserved areas (e.g., Giannini et al. 2015; Sawakuchi et al. 2013), as well as identifying possible resilient corridors between protected areas. In this context, restoration strategies can be adopted to increase connectedness in places that, as indicated in the landscape resilience classification scheme, would change the situation between classes from low to high local connectedness (from left to right): for example, directing actions at places indicated as class 14 so that, in the future, local connectedness increases and helps to improve the landscape by connecting these sites, which aids biological flow and consequently promotes the restoration of ecological processes (Tabor et al. 2019). Once the value changes to class 24 (the same example applies to class 24 to 34, and 34 to 44), it means that the outcomes of the restoration activities are maximized, potentially increasing vegetation structure and ecosystem functioning.

As mentioned before, our database can be used to identify areas for restoration as resilience to climate change is a relevant criterion to be used in the search for areas to be restored (Ravenscroft et al. 2010). We can prioritize different restoration strategies, such as relying on passive restoration in sites with intermediate to high landscape resilience, as these areas have higher values of both landscape heterogeneity and local connectedness, and since propagules or individuals from neighboring sites can more easily flow through the landscape and reach restoration sites. Therefore, higher local connectedness enables species to recolonize restoration sites (Tambosi et al. 2014), and landscape heterogeneity will allow their persistence in future conditions (Antonelli et al. 2018; Vernham et al. 2023). Conversely, active restoration strategies can be adopted to enhance connectivity on low‐resilient landscapes, further allowing the recovery of ecosystem services and the establishment of species that have already lost their habitat from disturbed landscapes (Pardini et al. 2018). Combined with climate data, this information can also be useful for defining more precise regions for the establishment of native species used in ecological restoration projects, as well as maps of the regeneration potential in all the six biomes (Ministério do Meio Ambiente [MMA] 2017).

## 5 Conclusion

Governments of megadiversity countries have the responsibility of managing their territories, by conserving or improving landscapes that are capable of providing for human needs compatible with biodiversity and ecosystem functioning. Climate change will likely make both tasks more difficult, since species and human resources are expected to change their distribution in space. Hence, long‐term planning considering such a dynamic scenario is quintessential. So far, most world prioritization exercises for conservation have been proposed considering a static climate scenario (e.g., biodiversity hotspots), and that is also true for more regional prioritization exercises (Oliveira et al. 2017). The results provided here have the potential to be used by decision‐makers at various spatial scales, from federal and state governments to local private enterprises and stakeholders. Landscape resilience information can be used for evaluating the existing network of protected areas and its expansion. Also, passive and active restoration actions can be designed more effectively considering the resilience information from this study. This map could contribute to the National Adaptation Plan to Climate Change (MMA 2016), which is currently under revision by the Brazilian government, and as a tool for the development of strategic actions for biodiversity conservation and land management. By using global databases coupled with national and transnational databases, our analysis allows for replication in different regions and contexts. To date, there has been no similar mapping of resilient sites for tropical regions. By emphasizing persistent abiotic features that shape ecological responses to climate change, our landscape resilience approach offers a robust and scalable tool that enriches frameworks centered on species traits, ecosystem dynamics, or planning under uncertainty. The methodology proposed here has the potential to be used in other megadiversity countries where challenges of climate change are pressing.

## Author Contributions

**Milena Fermina Rosenfield:** conceptualization, investigation, methodology, project administration, supervision, validation, visualization, writing – original draft, writing – review and editing. **Lucas Jardim:** data curation, formal analysis, investigation, methodology, validation, writing – original draft, writing – review and editing. **Marina Antongiovanni:** data curation, formal analysis, investigation, methodology, validation, writing – original draft, writing – review and editing. **Luciano Carramaschi de Alagão Querido:** data curation, formal analysis, investigation, methodology, validation, visualization, writing – original draft, writing – review and editing. **Alisson André Ribeiro:** data curation, formal analysis, investigation, methodology, validation, writing – original draft, writing – review and editing. **Andrea Sánchez‐Tapia:** data curation, formal analysis, investigation, methodology, validation, writing – original draft, writing – review and editing. **Priscila Silveira:** data curation, formal analysis, investigation, methodology, validation, writing – original draft, writing – review and editing. **Levi Carina Terribile:** investigation, methodology, validation, writing – original draft, writing – review and editing. **Eduardo M. Venticinque:** investigation, methodology, validation, writing – original draft, writing – review and editing. **Ana Luisa Albernaz:** investigation, methodology, validation, writing – original draft, writing – review and editing. **Letícia Couto Garcia:** investigation, methodology, validation, writing – original draft, writing – review and editing. **Leandro Reverberi Tambosi:** investigation, methodology, validation, writing – original draft, writing – review and editing. **Marcos Adami:** methodology, validation, writing – review and editing. **Fernando Gertum Becker:** methodology, validation, writing – review and editing. **Maíra Benchimol:** methodology, validation, writing – review and editing. **Luísa Gigante Carvalheiro:** methodology, validation, writing – review and editing. **Cintia Cornelius:** methodology, validation, writing – review and editing. **Geraldo Alves Damasceno‐Junior:** methodology, validation, writing – review and editing. **Ricardo Dobrovolski:** methodology, validation, writing – review and editing. **Manuel Eduardo Ferreira:** methodology, validation, writing – review and editing. **Carlos Roberto Fonseca:** methodology, validation, writing – review and editing. **José Guilherme Fronza:** methodology, validation, writing – review and editing. **Angela Terumi Fushita:** methodology, validation, writing – review and editing. **Adrian Antonio Garda:** methodology, validation, writing – review and editing. **Heinrich Hasenack:** methodology, validation, writing – review and editing. **Priscila Lemes:** methodology, validation, writing – review and editing. **Renata Libonati:** methodology, validation, writing – review and editing. **Camile Lugarini:** methodology, validation, writing – review and editing. **Marcia C. M. Marques:** methodology, validation, writing – review and editing. **Felipe Melo:** methodology, validation, writing – review and editing. **Alessandro Ribeiro de Morais:** methodology, validation, writing – review and editing. **Sandra Cristina Müller:** methodology, validation, writing – review and editing. **Andreza Viana Neri:** methodology, validation, writing – review and editing. **Rita de Cássia Quitete Portela:** methodology, validation, writing – review and editing. **Mario Barroso Ramos Neto:** methodology, validation, writing – review and editing. **Camila Linhares Rezende:** methodology, validation, writing – review and editing. **Fabio de Oliveira Roque:** methodology, validation, writing – review and editing. **Thadeu Sobral‐Souza:** methodology, validation, writing – review and editing. **Mariana M. Vale:** methodology, validation, writing – review and editing. **Gustavo M. Vasques:** methodology, validation, writing – review and editing. **Eduardo Vélez‐Martin:** methodology, validation, writing – review and editing. **Ima Vieira:** methodology, validation, writing – review and editing. **Fernanda P. Werneck:** methodology, validation, writing – review and editing. **Edenise Garcia:** conceptualization, funding acquisition, methodology, writing – review and editing.

## Conflicts of Interest

The authors declare no conflicts of interest.

## Supporting information


**Appendix S1:** gcb70544‐sup‐0001‐AppendixS1.docx.

## Data Availability

The data that support the findings of this study are openly available in Zenodo at https://doi.org/10.5281/zenodo.13320277.
