# Supplementary material for: Mapping Resilient Landscapes to Climate Change in a Megadiverse Country
Source: Glob Chang Biol. 2025 Oct 10;31(10):e70544. doi: 10.1111/gcb.70544 (PMC12512505; doi:10.1111/gcb.70544)
Supplement: Supplementary file 1 — Appendix S1: gcb70544‐sup‐0001‐AppendixS1.docx. [file GCB-31-e70544-s001.docx]

*Appendix A*

Mapping resilient landscapes to climate change in a megadiverse country

Milena Fermina Rosenfield, Lucas Jardim, Marina Antongiovanni, Luciano Carramaschi de Alagão Querido, Alisson André Ribeiro, Andrea Sánchez-Tapia, Priscila Silveira, Levi Carina Terribile, Eduardo M. Venticinque, Ana Luisa Albernaz, Letícia Couto Garcia, Leandro Reverberi Tambosi, Marcos Adami, Fernando Gertum Becker, Maíra Benchimol, Luísa Gigante Carvalheiro, Cintia Cornelius, Geraldo Alves Damasceno Junior, Ricardo Dobrovolski, Manuel Eduardo Ferreira, Carlos Roberto Fonseca, José Guilherme Fronza, Angela Terumi Fushita, Adrian Antonio Garda, Heinrich Hasenack, Priscila Lemes, Renata Libonati, Camile Lugarini, Marcia C. M. Marques, Felipe Melo, Alessandro R. Morais, Sandra Cristina Müller, Andreza Viana Neri, Rita de Cássia Quitete Portela, Mario Barroso Ramos Neto, Camila Linhares Rezende, Fabio de Oliveira Roque, Thadeu Sobral-Souza, Mariana M. Vale, Gustavo M. Vasques, Eduardo Vélez-Martin, Ima Vieira, Fernanda P. Werneck, Edenise Garcia. *Global Change Biology.*

# 1 Methods

# 1.1 Study area

The terrestrial biomes present in Brazil are shown in Fig. A1: Amazônia in the north; Caatinga in the northeast; Cerrado in the center; Mata Atlântica along the coastline; Pampa in the south; and Pantanal in west.


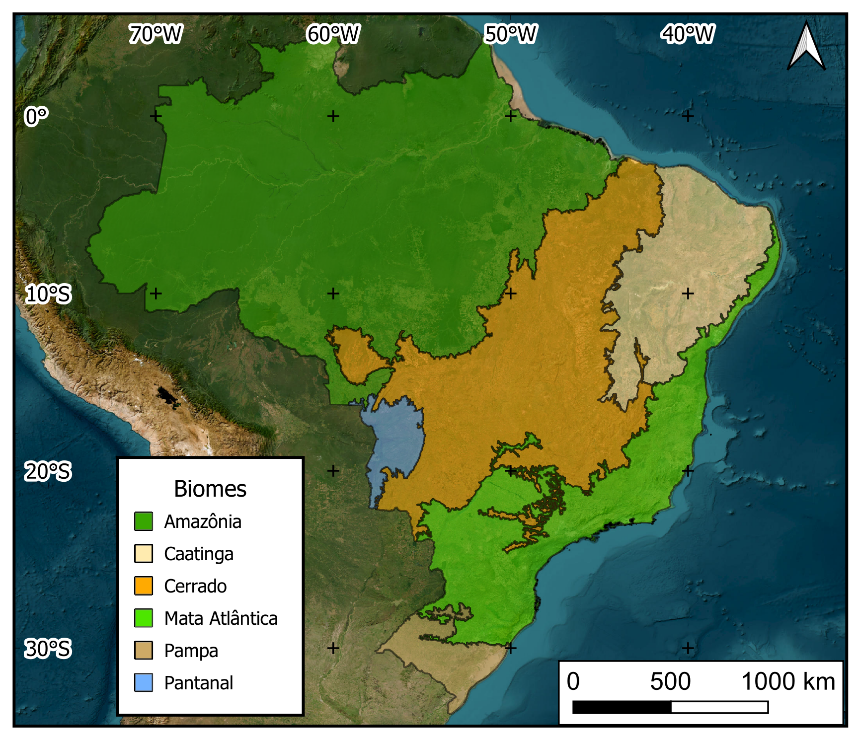


Figure A1. Map of the six terrestrial biomes in Brazil.

# 1.2 Landscape heterogeneity

# 1.2.1 Databases

We used the Merit-DEM digital elevation model (DEM) (Yamazaki et al., 2017), at 90 m spatial resolution, as the basis for terrain calculations such as slope, aspect and topographic position index. This DEM is a product on a global scale, allowing the analysis to be replicated in other regions, and has corrections for various biases derived from radar images, especially in areas with a high density of forests such as the Amazon rainforest. The purpose of these corrections is to transform the data from a digital surface model (which considers the objects above the terrain) into a digital terrain model (which considers the elevation at ground level).

In addition, Merit-DEM already has a global-scale flux accumulation layer available in Merit-Hydro (Yamazaki et al., 2019). This flow accumulation layer has corrections for flat areas and for the effect of tree density in the calculation of the hydrographic network (Yamazaki et al., 2019), which are important for the analysis of tropical forests with high tree density. However, flow accumulation does not adequately capture the distribution and area of lakes and wide rivers, such as the Amazon River. We therefore included class 33 from MapBiomas (MapBiomas Project, 2023), which represents rivers and lakes, to complement the information on wetlands. MapBiomas is a national project to map and classify land use changes over the last 37 years (1985 to 2022), using remote sensing data.

# 1.2.2 Generating variables

Landscape heterogeneity is calculated using hierarchical averages of the Z values of four different variables: landform variety, elevation range, wetland score and soil richness. Below we describe how each variable that makes up the metric is created and how they are combined to form landscape heterogeneity.

Landform variety

Landforms represent the variation in humidity, exposure to solar radiation, wind speed and sediment deposition in the landscape (Anderson et al., 2016; Dobrowski, 2011). This classification is determined by the variables slope, aspect, topographic position index, moisture index and the distribution of rivers and lakes. The combination of these variables makes it possible to identify mountain tops and valleys, steep slopes or flat areas, relief exposure with more shade or sunlight, dry or wet areas due to flow accumulation, and the presence of lakes and rivers (Fig. A2). The classification was based on previous studies (Anderson et al., 2016, 2014, 2023; Fels and Matson, 1996) conducted for North America (https://crcs.tnc.org/pages/land), with a few modifications to adjust the methods to the characteristics of our study region as described below.


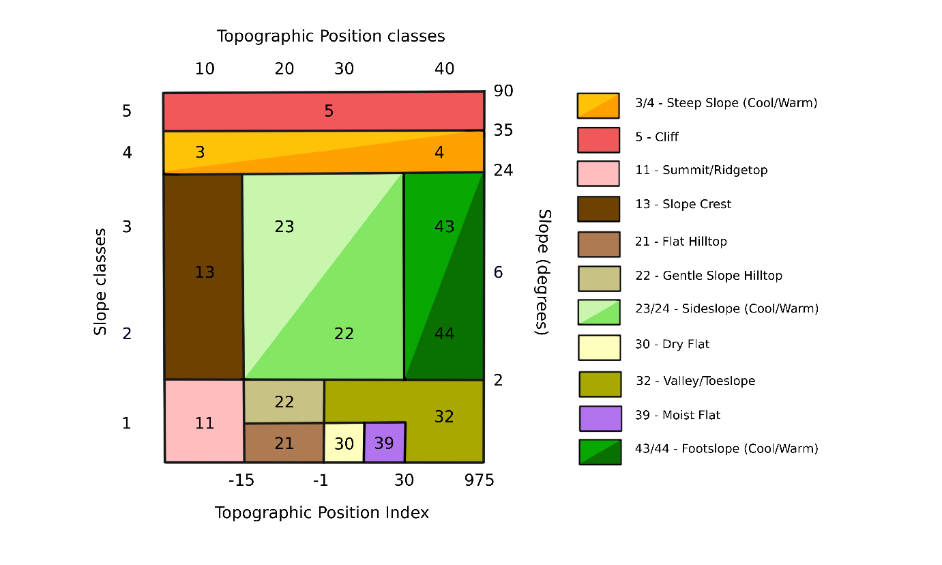


Figure A2. Classification of landforms according to slope, aspect, topographic position index, moisture index, rivers and lakes. Modified from Anderson et al. (2016).

We used the following procedures to calculate each variable of used to estimate the landform variety:

- *Slope*: calculated using the ee.Terrain.slope function in Google Earth Engine, as a local gradient of the four adjacent cells. The results are presented in degrees of slope (0º to 90º).
- *Aspect*: calculated using the ee.Terrain.aspect function in Google Earth Engine, as a local gradient of the four adjacent cells. The results are presented in degrees of relief direction (0º = north, 90º = east, 180º = south and 270º = west). We divided the aspect into two groups based on the amount of solar incidence, with cells with values between 90º and 270º classified as cold faces and those with values between 0º to 90º and 270º to 360º classified as hot faces.
- *Topographic position index (TPI)*: calculated on three scales using a circular window with 7, 11 and 15 radius cells. It was calculated from the sum of the difference between the elevation of the focal cell and its neighbors (*i*), divided by the number of neighboring cells (*n*), following the formula: $TPI=\frac{\sum_{i}^{n} \left( {neighbors}_{i}-focal \right)}{n}$. The index is composed of the average TPI of the three scales, which allows the consideration of local and regional levels of landscape resolution (Theobald et al., 2015). This approach was implemented to enable the classification of landforms that emerge at both local (e.g. valleys and mountain tops) and regional (e.g. flat plateau tops; Fels and Matson, 1996) scales. The window sizes were visually adjusted to represent the landforms.
- *Moisture index*: calculated based on the flow accumulation, generated from Merit-Hydro, and the slope, calculated previously, using the following formula: $moisture.index=\frac{log(flow+1)}{(slope+1)}\times1000$. After calculating the moisture index for each cell, we smoothed the distribution pattern of the drainage network as the average of the index within a circular window with a radius of one cell.

Each index (slope, aspect, TPI and moisture index) was transformed into classes to form landform types (Fig. A2). The slope and aspect followed the classification in Anderson et al. (2016), with aspect adjusted for the Southern Hemisphere. The TPI and moisture index thresholds were set visually. We only classified as moist areas cells with a moisture index above 3,000, since lower values overestimated the distribution of water bodies in flat areas. Finally, we combined the wetlands map with the water and lakes map from MapBiomas.

The classes of each variable were combined to represent the landforms as a numerical code: humidity index * 1000; landform exposure * 100; TPI * 10; landform slope * 1. For example, code 11 (0011) represents the first slope class (areas of low slope) and the first TPI class (landform position higher than the surrounding area), and is therefore a summit. Examples of landforms identified in Brazil, considering different biomes can be seen in Fig. A3.


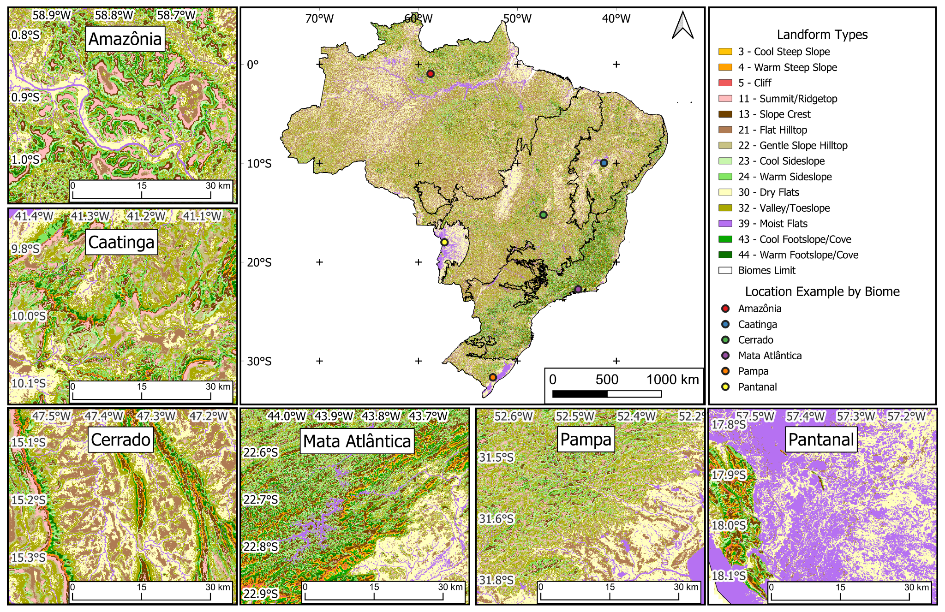


Figure A3. Landform types mapped for Brazil and examples for each biome.

After classifying landform types, landform variety was calculated as the number of landforms within a circular window of the focal cell (Fig. A4). The size of the window radius was defined by calculating the variety at different radii (2, 5, 7, 10, 15 and 20 cells) and calculating the difference in the average variety obtained for Brazil with each increase in radius. The radius chosen was the one at which the subsequent one did not add variation. In this way, the radius represents the level of landscape resolution that captures the maximum variety of landforms, and the value of 5 radius cells (450 m) was chosen for the whole of Brazil. The same procedure was conducted for the additional variables (elevation range, wetland score and soil richness).


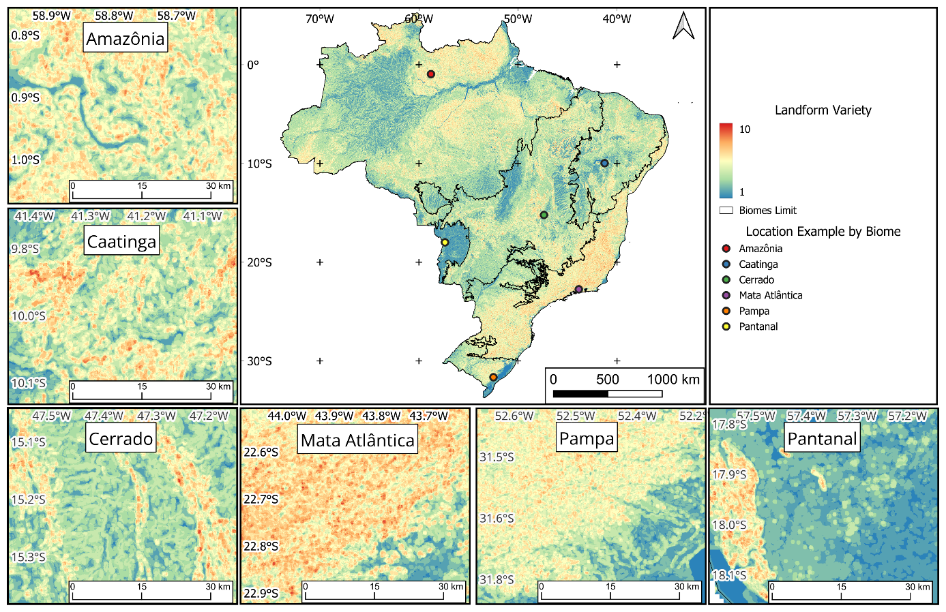


Figure A4. Landform variety mapped for Brazil and examples for each biome.

Finally, the values for landform variety were standardized in order to compose landscape heterogeneity (Fig. A5). The standardization used a moving window with a radius of 200 pixels, where we calculated the average of the neighborhood ($\mu_{neighborhood}$) of the cell value ($X_{cell}$) and the standard deviation of the neighborhood ($\sigma_{neighborhood}$). The Z-score was calculated by subtracting the cell values by the average of their neighborhood and dividing by the standard deviation: $Z_{cell}=\frac{X_{cell}-\mu_{neighborhood}}{\sigma_{neighborhood}}$ .


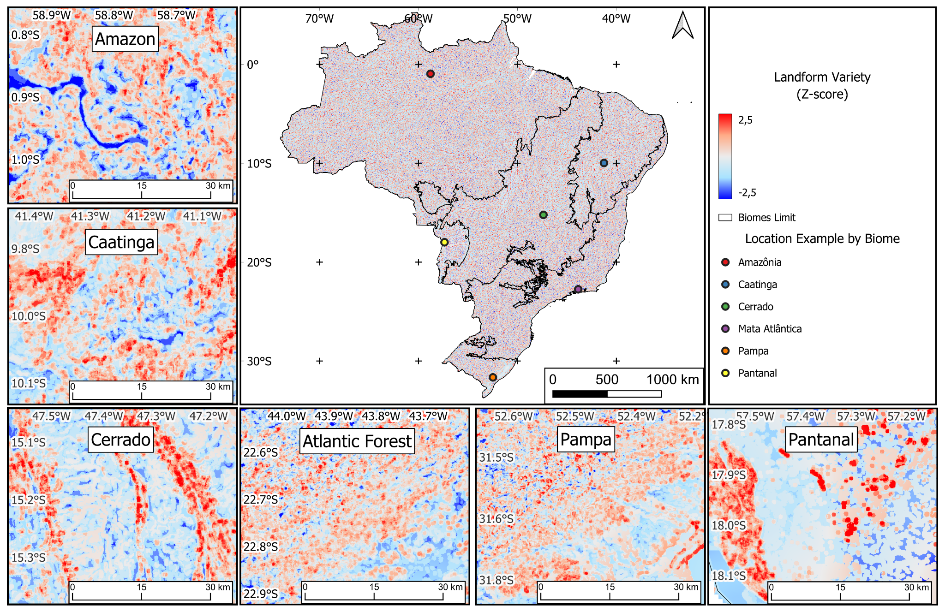


Figure A5. Landform variety standardized (Z-score) calculated for Brazil and examples for each biome.

Elevation range

The elevation range represents the variation in altitude in a region, regardless of the variety of landforms, and was calculated as the difference between the maximum and minimum altitude values within a circular window of 450 m (5 cell radius), using MERIT-DEM (Yamazaki et al., 2017). The size of the window was selected following the same procedure conducted for landform variety (see above). We then performed a Simple Linear Regression (Ordinary Linear Regression) between the elevation range values and landform variety and obtained the residual values from this analysis as the elevation range independent of landform variety (Fig. A6). The elevation range values were also standardized in Z-scores (as described above; Fig. A7).


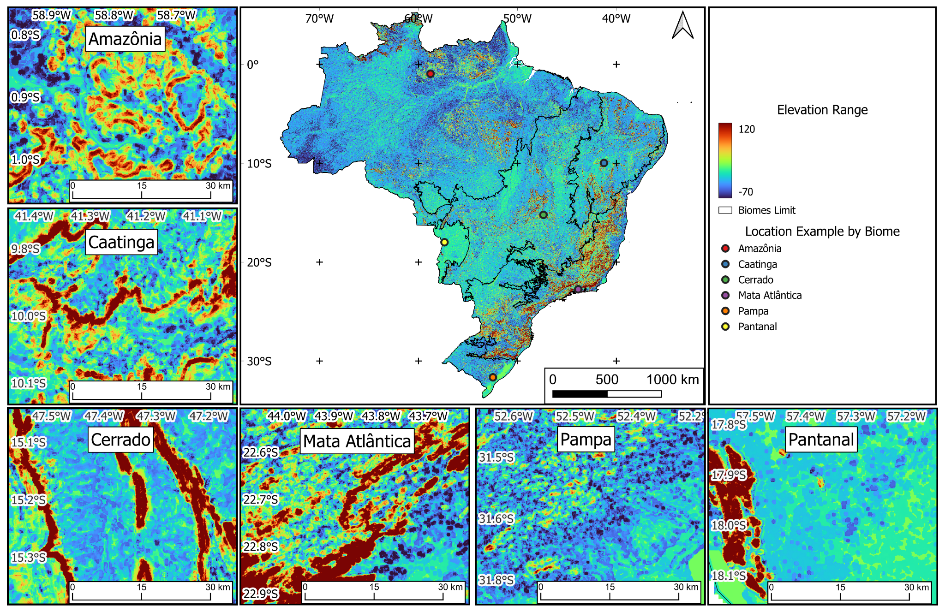


Figure A6. Elevation range mapped for Brazil and examples for each biome.


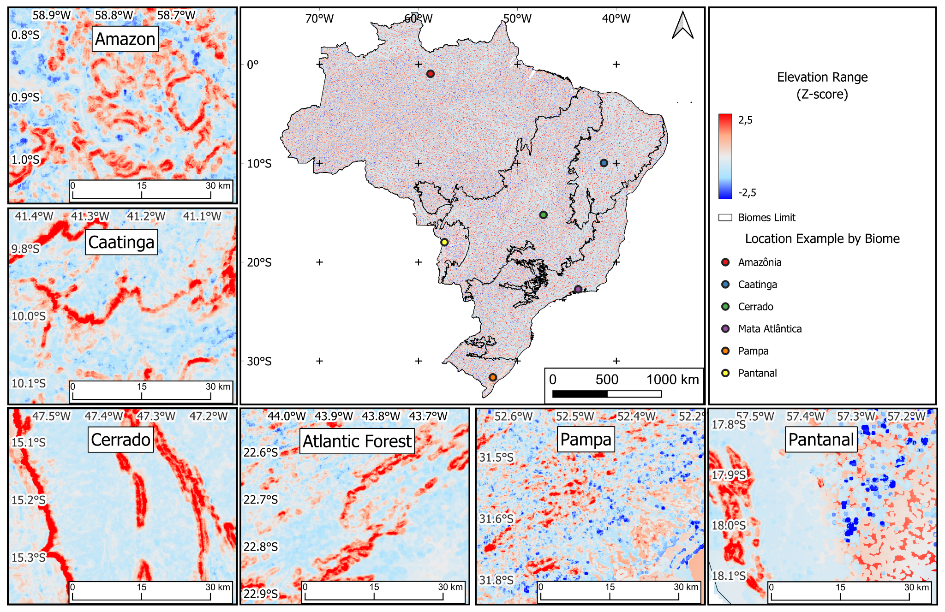


Figure A7. Elevation range (Z-score) mapped for Brazil and examples for each biome.

Wetland score

The wetland score was calculated using data from the Global Wetlands Database (Gumbricht et al., 2017). This database provides information and inventory of wetlands worldwide. The data is obtained from satellite images, aerial sampling and published reports. In this study, we resampled the wetlands map from 124 m to 90 m, the same resolution used to the other variables. We then calculated the wetland score considering the density of wetlands at the local scale (450 m) and at the regional scale (1,170 m), and wetland patchiness, defined as the number of regional wetlands (Anderson et al., 2016), as described below. Local and regional scales were defined following the methodology proposed previously (Anderson et al., 2016, 2023).

First, we calculated the number of wetlands as the number of cells classified as wetlands within a local (450 m) and regional (1170 m) window. Dividing the number of wetlands by the number of cells in the window generates the local and regional density of wetlands. Local and regional wetland densities and the regional wetland quantity were then transformed into Z-scores. The mean ($\mu_{neighborhood}$) and standard deviation ($\sigma_{neighborhood}$) were calculated within a circular neighborhood of 200 cells radius (18 km) of each focal cell ($X_{cell}$).

The final wetland score was calculated as the weighted average of the Z-score of local ($Z_{local.density}$) and regional density ($Z_{regional.density}$), giving double weight to local density: $Z_{wetland.score}=\frac{(Z_{local.density}\times2)+Z_{regional.density}}{3}$. For the cells where the amount of regional wetlands ($Z_{regional.count}$) was greater than the wetland score, the index was calculated as the weighted average of the densities and the amount of wetlands, again with a double weight to local density (Fig. A8), using the following formula: $Z_{wetland.score}=\frac{(Z_{local.density}\times2)+Z_{regional.density}+Z_{regional.count}}{3}$.


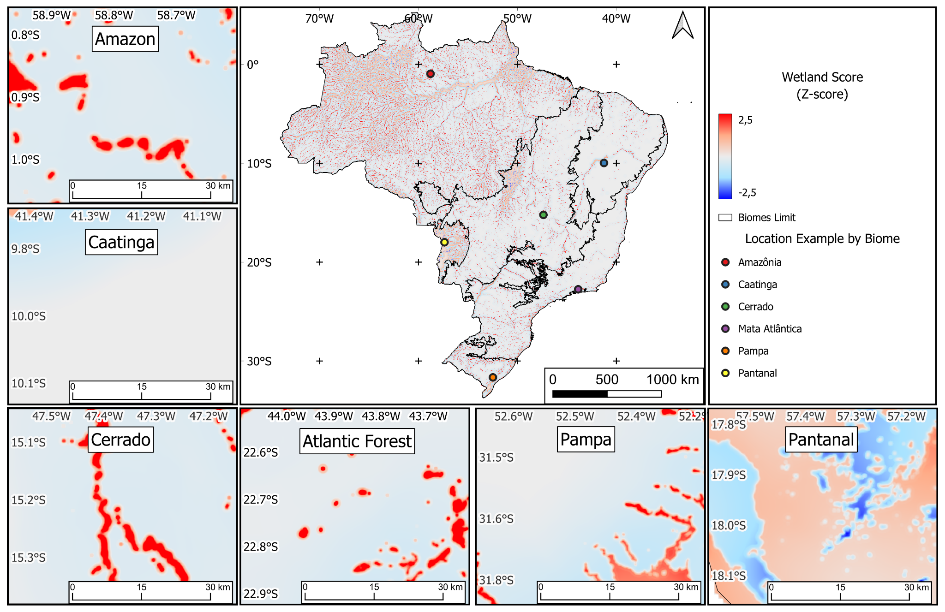


Figure A8. Wetland score standardized (Z-score) calculated for Brazil and examples for each biome.

Soil richness

Soil richness was calculated as the total number of dominant and subdominant soil types (Fig. A9) found within the soil polygons obtained from the shapefile produced by the Brazilian Institute of Geography and Statistics (IBGE) (<https://www.ibge.gov.br/geociencias/informacoes-ambientais/pedologia/10871-pedologia.html>). We counted and summed all the different classes at each feature recorded in the columns “*component*”, “*component1*”, “*component2*”, and “*component3*” of the shapefile’s attribute table with the field calculator in QGIS. This information was rasterized and projected at the same spatial resolution as the aforementioned variables (Fig. A9), and then standardized in Z-scores (as described above; Fig. A10).


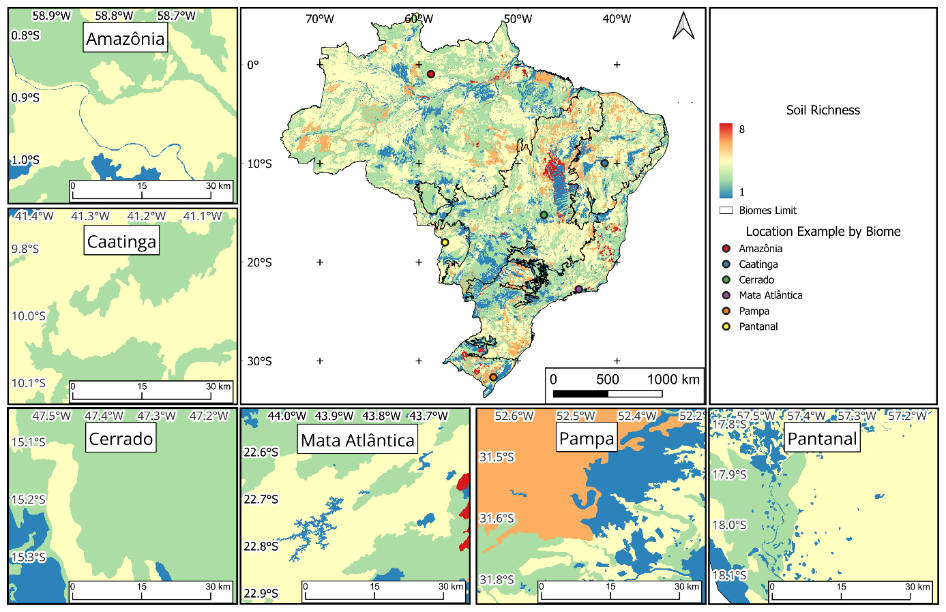


Figure A9. Soil richness mapped for Brazil and examples for each biome.


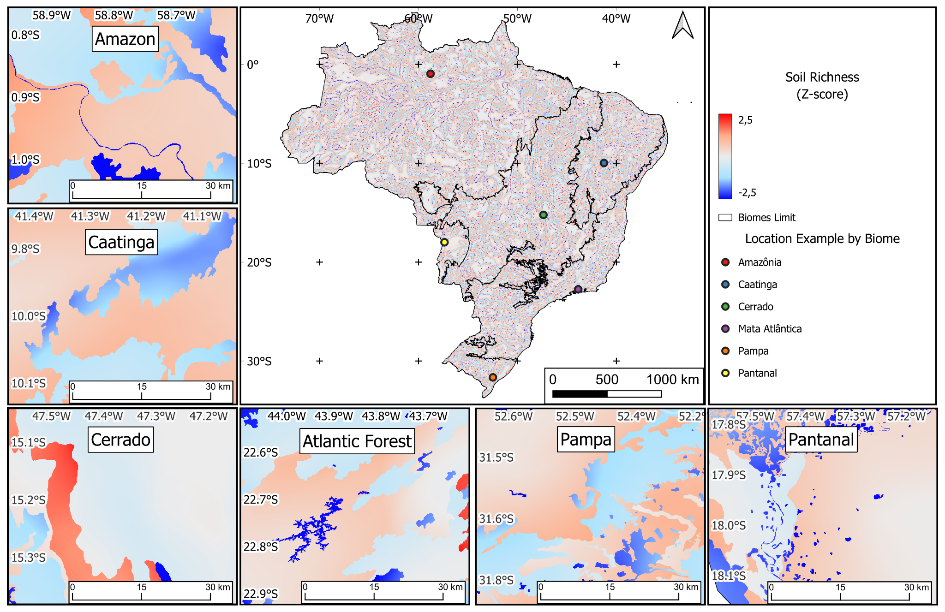


Figure A10. Soil richness standardized (Z-score) calculated for Brazil and examples for each biome.

# 1.2.3 Calculating landscape heterogeneity

Landscape heterogeneity was calculated following a hierarchical approach that considers the additional effect of the following variable in the previous one. The calculation follows an evaluation by the order of importance of each variable in sustaining a higher biodiversity in the area. So the relevance of a variable was determined by its value pixel-by-pixel, firstly with landform variety, then elevation range, wetland score and finally soil richness. Thus, the relevance of a variable for landscape heterogeneity is determined by which variable had the higher value in the cell when compared with the other variables used to create the average z-score for the final product. We considered the following steps to generate the layer of landscape heterogeneity:

- In step 1, landscape heterogeneity was defined as the Z-score of landform variety.
- In step 2, in places where the Z-score for elevation range was greater than that obtained for landform variety, landscape heterogeneity was calculated as the weighted average of the two variables, giving double weight to landform variety, using the following formula: $Z_{landscape.heterogeneity}=\frac{(Z_{landform}\times2)+Z_{elevation}}{3}$.
- In step 3, in places where the wetland score was higher than the landscape heterogeneity calculated in step 2, we calculated the weighted average of landscape heterogeneity and wetland score, giving wetlands double the weight, using the following formula: $Z_{landscape.heterogeneity}=\frac{Z_{landform}+Z_{elevation}+(Z_{wetland.score}\times2)}{4}$. The double weighting of wetlands is justified by the fact that these sites are in flat areas with low topographic variability, and wetlands are places with high water availability that influence microclimate variability.
- If, in step 2, the elevation range was not relevant for the cell, landscape heterogeneity in step 3 was calculated using the following formula: $Z_{landscape.heterogeneity}=\frac{Z_{landform}+(Z_{wetland.score}\times2)}{3}$.
- In step 4, where the Z-score for soil richness was greater than the landscape heterogeneity calculated in step 3, the values were replaced by the weighted average of the variables in that location, with double the weight for landform variety, using the following formula: $Z_{landscape.heterogeneity}=\frac{(Z_{landform}\times2)+Z_{elevation}+Z_{wetland.score}+Z_{soil.richness}}{5}$.
- If, in step 3, the wetland score was not relevant to a particular cell, landscape heterogeneity was calculated using the following formula: $Z_{landscape.heterogeneity}=\frac{(Z_{landform}\times2)+Z_{elevation}+Z_{soil.richness}}{4}$. Similarly, in places where the elevation range was not relevant to a particular cell, landscape heterogeneity was calculated using the following formula: $Z_{landscape.heterogeneity}=\frac{(Z_{landform}\times2)+Z_{wetland.score}+Z_{soil.richness}}{4}$.
- Finally, in places where only landform variety was relevant, landscape heterogeneity was calculated using the following formula:$Z_{landscape.heterogeneity}=\frac{(Z_{landform}\times2)+Z_{soil.richness}}{3}$.

# 1.3 Local connectedness

# 1.3.1 Databases

Local connectedness was calculated based on land use and land cover data provided by MapBiomas, for the year 2021 (MapBiomas Project, 2023). We supplemented this land use and land cover layer with information on river width, transportation infrastructure and energy infrastructure:

- River width was derived from the open water layers from Brazil (MapBiomas Project, 2023), the river basins at level 8 (Lehner and Grill, 2013) and the effective width of water bodies (Yamazaki et al., 2014);
- Transportation infrastructure was obtained from the layers of paved roads, unpaved roads and railroads provided by the Brazilian Institute of Geography and Statistics (IBGE - BCIM250, available at <https://www.ibge.gov.br/geociencias/cartas-e-mapas/bases-cartograficas-continuas/15759-brasil.html?=&t=downloads>);
- Energy infrastructure correspond to the layers of wind turbines, photovoltaic power plants, wind power transmission lines and thermoelectric power plants provided by the National Electric Energy Agency (ANEEL, available at https://gisepeprd2.epe.gov.br/WebMapEPE/) and power transmission lines provided by the Brazilian Institute of Geography and Statistics (IBGE - BCIM250, available at <https://www.ibge.gov.br/geociencias/cartas-e-mapas/bases-cartograficas-continuas/15759-brasil.html?=&t=downloads>).

# 1.3.2 Steps of the analysis

Resistance values are relative measures of the difficulty that different types of land use and land cover potentially confer on the movement of organisms in the landscape. These values were assigned by biome, following two assumptions: (a) the greater the structural difference between a given type of land cover and the native local vegetation present in the biome, the greater the resistance value of the land cover class; and (b) wider bodies of water offer greater resistance to the movement of terrestrial organisms across the landscape than narrower bodies of water.

Surface resistance is based on a land use and land cover data layer complemented with information on river width, transportation infrastructure and energy infrastructure. A flowchart showing the stages of the local connectedness analysis is provided in Fig. A11. There are mainly five stages, encompassing: reclassification of the spatial resolution (stage 1); incorporating width of water bodies (stage 2) and transportation and energy infrastructure databases (stage 3); assigning resistance values and combining databases (stage 4); and applying *kernel* filter and generating final layer (stage 5). Details of each stage are provided below.


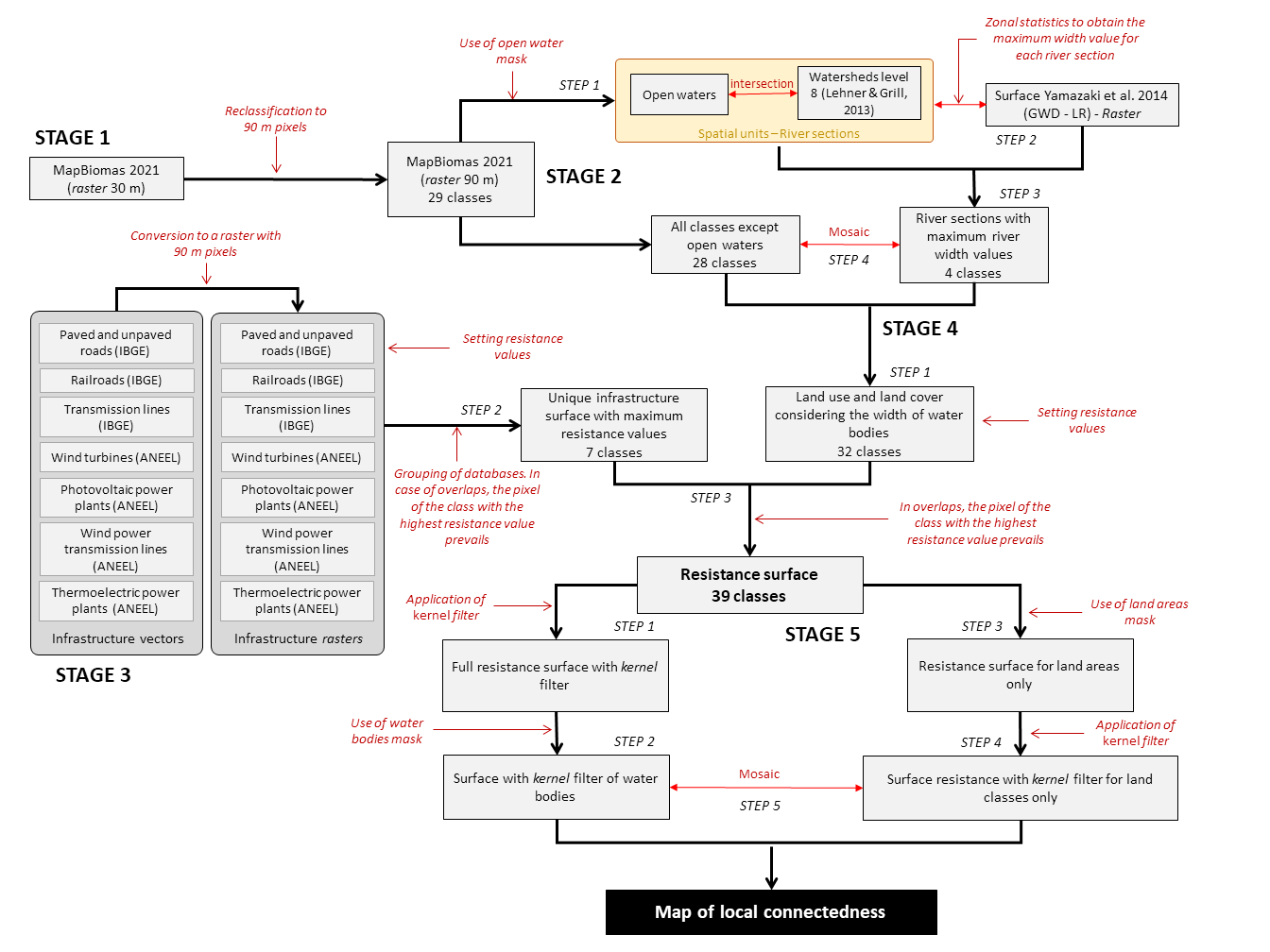


Figure A11. Flowchart showing the stages of the local connectedness analysis.

In stage 1 of the analysis, we resampled the MapBiomas data (MapBiomas Project, 2023) from the original 30 m resolution to 90 m resolution, in order to use the same 90 m spatial resolution assigned for the landscape heterogeneity layer. This stage generated a total of 29 land use and land cover classes.

In stage 2, we determined the effect of river widths to landscape resistance, since wider rivers tend to be stronger barriers to the movement of organisms than smaller rivers (Hayes and Sewlal, 2004). We evaluated the maximum size of the body of water regionally using the MapBiomas class equivalent to open waters (rivers, lakes, and oceans). We subdivided this layer into four distinct classes according to the width of the bodies of water. To obtain these subclasses, we extracted the mask of water bodies and divided this single class into smaller pieces, regionalizing their widths by sections. For each of these sections, we extracted the maximum effective width of the water bodies, from bank to bank, using the GWD - LR base provided by Yamazaki et al. (2014). The sections were grouped into the following width classes: (1) 1 to 250 m; (2) 250 to 1,000 m; (3) 1,000 to 4,000 m; and (4) greater than 4,000 m. Combining these four classes with the land use and land cover classes mentioned above (minus the water bodies class), generated a total of 32 classes.

In stage 3, we combined transportation and energy infrastructure layers to the map generated in the previous stage. These databases were converted from their original vector format to matrix or raster format, with pixels of 90 m in size. This stage generated a total of 7 classes, which combined with the previous stage generated a total of 39 classes.

In stage 4, we assigned resistance values and combined the databases. Each of the land use and land cover classes in MapBiomas plus the four water body width classes (32 classes), was assigned a resistance value per biome based on the assumptions set out above regarding the structural difference between land use and the type of vegetation characteristic of the biome. Resistance values ranged from 1 (low resistance; e.g. natural vegetation, forest, grassland) to 20 (high resistance; e.g. urban and mining areas) and were assigned by technical experts from the study (Table A1). The different classes from transportation and energy infrastructure were also given specific resistance values (as shown in Table A1) and all separate layers were grouped into a single infrastructure raster. They were combined with the previous layer in a way that when pixels from different classes overlapped, the value of the pixel with the highest resistance always prevailed. The combination of these layers resulted in a single resistance surface (Fig. A12).

Table A1. Resistance values for the 39 land use and land cover classes. The classes are the result of the classes from MapBiomas (N: 1 - 28), river size classes (N: 29 - 32), and transportation and energy infrastructures (N: 33 - 39). Not all classes are present in every biome, so missing values indicate that the class was not present in the biome.

| **N** | **Land use and land cover classes** | **Amazônia** | **Caatinga** | **Cerrado** | **Mata Atlântica** | **Pampa** | **Pantanal** |
| --- | --- | --- | --- | --- | --- | --- | --- |
| 1 | Flooded forest | 1 |  |  |  |  |  |
| 2 | Mangrove | 1 | 1 | 1 | 1 |  |  |
| 3 | Forest | 1 | 1 | 1 | 1 | 1 | 1 |
| 4 | Wetland | 1 |  | 1 | 2 | 1 | 1 |
| 5 | Savanna | 1 | 1 | 1 | 2 |  | 1 |
| 6 | Grassland | 1 | 1 | 1 | 2 | 1 | 1 |
| 7 | Wooded sandbank vegetation |  | 1 |  | 1 | 1 |  |
| 8 | Herbaceous sandbank vegetation |  | 1 |  | 2 | 1 |  |
| 9 | Salt flat | 2 | 1 | 2 | 2 |  |  |
| 10 | Beach, dune and sand spot | 2 | 2 | 2 | 2 | 2 | 2 |
| 11 | Rocky outcrop | 3 | 1 | 2 | 3 | 2 |  |
| 12 | Other non forest formation |  | 7 |  | 2 |  |  |
| 13 | Cotton | 7 | 7 | 10 |  |  |  |
| 14 | Silviculture (forest plantation) | 7 | 2 | 7 | 3 | 10 | 5 |
| 15 | Dendê | 10 |  |  |  |  |  |
| 16 | Citrus |  |  | 10 | 4 |  |  |
| 17 | Coffee |  | 7 | 10 | 4 |  |  |
| 18 | Other perennial crops | 10 | 7 | 7 | 4 |  |  |
| 19 | Aquaculture |  | 10 | 7 | 7 | 7 |  |
| 20 | Rice |  |  | 10 | 10 | 10 |  |
| 21 | Mosaic of uses | 10 | 7 | 10 | 10 | 10 | 7 |
| 22 | Other non vegetated areas | 10 | 10 | 10 | 10 | 10 | 10 |
| 23 | Sugar cane | 15 | 10 | 10 | 10 |  | 10 |
| 24 | Other temporary crops | 15 | 7 | 10 | 10 | 10 | 5 |
| 25 | Pasture | 17 | 7 | 7 | 9 | 7 | 7 |
| 26 | Soybean | 17 | 10 | 10 | 10 | 10 | 10 |
| 27 | Urban Area | 20 | 20 | 20 | 20 | 20 | 20 |
| 28 | Mining | 20 | 20 | 20 | 20 | 20 | 20 |
| 29 | Water body up to 250 m | 2 | 2 | 2 | 2 | 2 | 2 |
| 30 | Water body from 250 to 1,000 m | 5 | 5 | 5 | 5 | 5 | 5 |
| 31 | Water body from 1,000 to 4,000 m | 10 | 10 | 10 | 10 | 10 | 10 |
| 32 | Water body over 4,000 m | 12 | 12 | 12 | 12 | 12 | 12 |
| 33 | Transmission line | 10 | 7 | 7 | 7 | 7 | 7 |
| 34 | Unpaved road | 10 | 10 | 7 | 10 | 7 | 3 |
| 35 | Railroad | 10 | 10 | 10 | 10 | 10 | 10 |
| 36 | Wind turbine | 15 | 15 | 15 | 15 | 15 | 15 |
| 37 | Photovoltaic power plant | 15 | 17 | 17 | 17 | 17 | 17 |
| 38 | Paved road | 20 | 20 | 20 | 20 | 20 | 20 |
| 39 | Thermoelectric power plant | 20 | 20 | 20 | 20 | 20 | 20 |


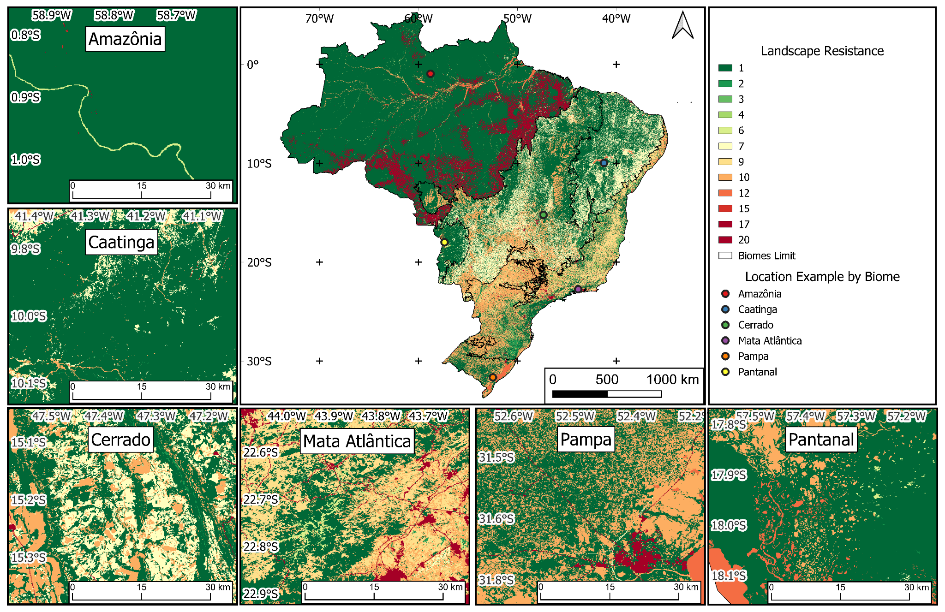


Figure A12. Map of surface resistance for Brazil, ranging from low resistance (1) to high resistance (20), according to different types of land use and land cover, river width classes, and transportation and energy infrastructures.

In stage 5, we generated the final local connectedness layer by applying a linear decay kernel filter (Fig. A13) to the landscape resistance surface obtained. This analysis considered, in a circular moving window of 23 pixels (approximately 2,070 m; Fig. A13), the spatial context in which each pixel is inserted, recognizing that closer pixels have a greater influence than more distant ones. The size of the window was determined following Anderson et al. (2014). By doing this, the kernel function helps in the task of finding the best travel paths in the landscape, i.e. those paths that offer the least potential resistance to species movement, based on land use and the local context of the landscape.


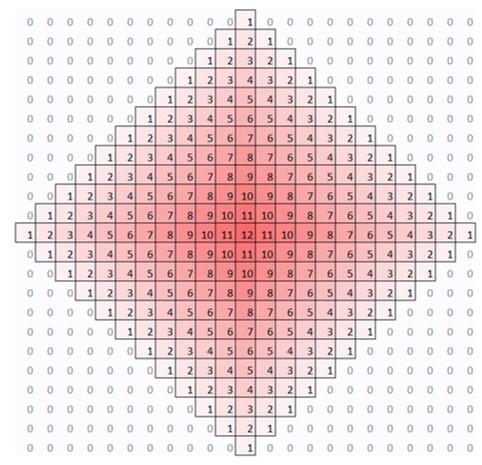


Figure A13. Visualization of the linear decay kernel filter, with 23 x 23 pixels, used in the analysis.

An important consideration is that, since our analysis was directed towards terrestrial organisms and the kernel function considers the effect of context when calculating local connectivity, we chose to disregard the effect that water pixels have on terrestrial pixels in the neighborhood analysis, but kept the opposite effect. In practical terms, this means that the cost of moving a terrestrial organism in a native forest pixel, for example, which is located on the edge of a wider body of water, should not be greater than the cost of moving in a forest pixel which is located on the edge of a narrow body of water. On the other hand, if a small body of water is located in an area of low resistance, such as a forest formation, it will have higher local connectivity values than if it was located in an area of high resistance, such as a pasture, for example.

# 1.4 Landscape resilience

To calculate landscape resilience, we used the landscape heterogeneity and local connectedness layers described above. Based on these two layers, we classified the images into quartiles based on the histogram of the distribution of the values of these metrics (Fig. A14).


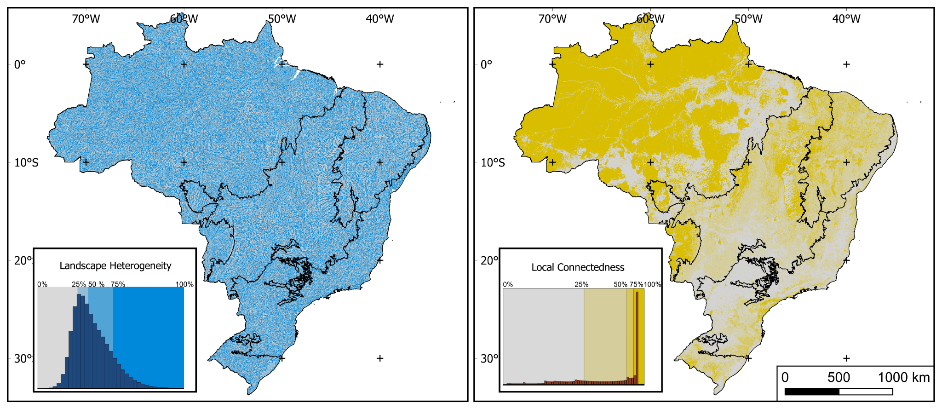


Figure A14. Classification of the histogram of pixel values in quartiles for landscape heterogeneity and local connectivity.

With the overlap of the two images classified by quartiles, we generated a combination of classes (11 - 44) for landscape resilience, which aims to show the areas where the results of the two metrics overlap or diverge (Fig. A15).


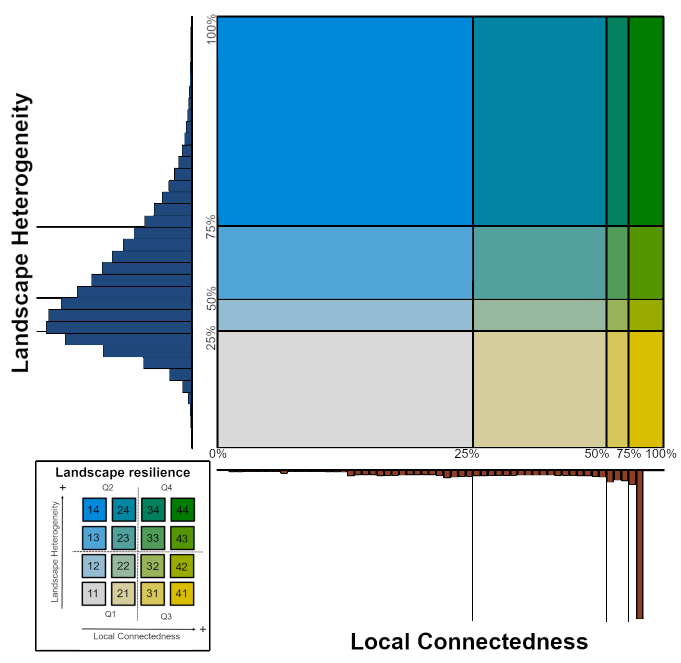


Figure A15. Definition of landscape resilience classes, based on the histogram of pixel distribution in the original landscape heterogeneity and local connectedness images.

# 2 Results

# 2.1 Landscape heterogeneity

The contribution of the different components of landscape heterogeneity (i.e. landform variety, elevation range, wetland score, and soil richness) across Brazil are shown in Fig. A16.


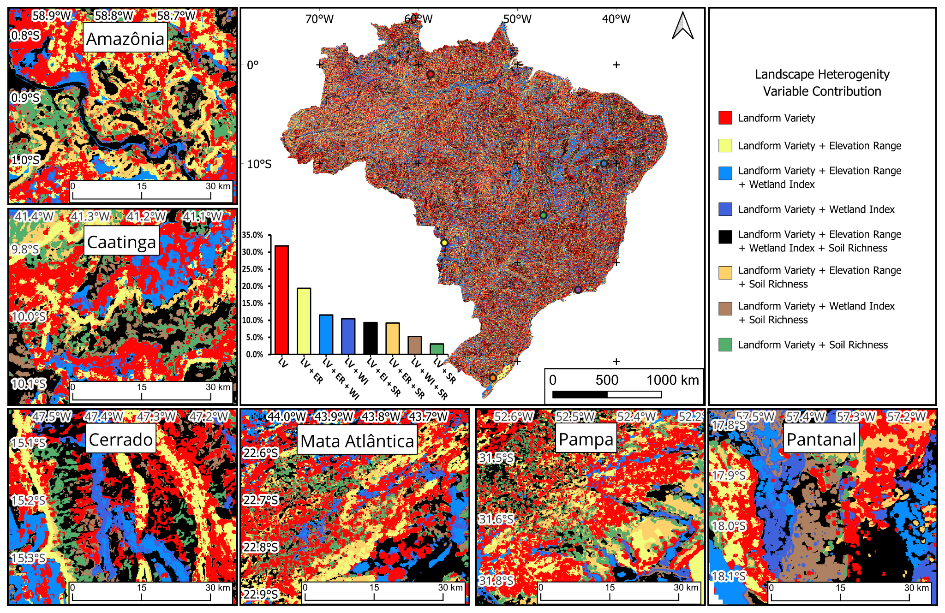


Figure A16. Relative contribution of each combination of variables that make up landscape heterogeneity. The map of Brazil and biome shots show the spatial distribution and regions where every combination has the most contribution and the bar graph shows the percentage of cells for every combination. The variables are landform variety (LV), elevation range (ER), wetland index (WI) and soil richness (SR). Landscape heterogeneity is represented as the weighted average of the z-scores of all variables.

# 3 Tutorials

The tutorials, showing the step-by-step analysis in Google Earth Engine and ArcGIS, for the layers and maps used in this manuscript can be found in the following links:

1. *Landscape heterogeneity:* <https://resiliencia-climatica-brasil.github.io/diversity-resilience-python/apresentacao.html>
2. *Local connectedness:* <https://resiliencia-climatica-brasil.github.io/Resistencia/>
3. *Landscape resilience:* <https://resiliencia-climatica-brasil.github.io/diversity-resilience-python/resilience.html>

# References

Anderson, M., Barnett, A., Clark, M., Prince, J., Olivero, S. A., and Vickery, B.: Resilient and Connected Landscapes for Terrestrial Conservation, 2016.

Anderson, M. G., Clark, M., and Sheldon, A. O.: Estimating Climate Resilience for Conservation across Geophysical Settings, Conserv. Biol., 28, 959–970, https://doi.org/10.1111/cobi.12272, 2014.

Anderson, M. G., Clark, M., Olivero, A. P., Barnett, A. R., Hall, K. R., Cornett, M. W., Ahlering, M., Schindel, M., Unnasch, B., Schloss, C., and Cameron, D. R.: A resilient and connected network of sites to sustain biodiversity under a changing climate, PNAS, 120, e2204434119, https://doi.org/10.1073/pnas.2204434119, 2023.

Dobrowski, S. Z.: A climatic basis for microrefugia: The influence of terrain on climate, Glob. Chang. Biol, 17, 1022–1035, https://doi.org/10.1111/j.1365-2486.2010.02263.x, 2011.

Fels, J. E. and Matson, K. C.: A cognitively-based approach for hydrogeomorphic land classification using digital terrain models., Third International Conference/Workshop on Integrating GIS and Environmental Modeling, Santa Fe, New Mexico, USA, 1996.

Gumbricht, T., Román-Cuesta, R. M., Verchot, L. V., Herold, M., Wittmann, F., Householder, E., Herold, N., and Murdiyarso, D.: Tropical and subtropical wetlands distribution version 2, https://doi.org/10.17528/cifor/data.00058, 2017.

Hayes, F. E. and Sewlal, J. N.: The Amazon River as a dispersal barrier to passerine birds: effects of river width, habitat and taxonomy, J. Biogeogr., 31, 1809–1818, https://doi.org/10.1111/j.1365-2699.2004.01139.x, 2004.

Lehner, B. and Grill, G.: Global river hydrography and network routing: baseline data and new approaches to study the world’s large river systems, Hydrol. Process., 27, 2171–2186, https://doi.org/10.1002/hyp.9740, 2013.

MapBiomas Project: Collection 8 of the Annual Series of Land Use and Land Cover Maps of Brazil, 2023.

Theobald, D. M., Harrison-Atlas, D., Monahan, W. B., and Albano, C. M.: Ecologically-Relevant Maps of Landforms and Physiographic Diversity for Climate Adaptation Planning, PLOS ONE, 10, e0143619, https://doi.org/10.1371/journal.pone.0143619, 2015.

Yamazaki, D., O’Loughlin, F., Trigg, M. A., Miller, Z. F., Pavelsky, T. M., and Bates, P. D.: Development of the Global Width Database for Large Rivers, Water Resour. Res., 50, 3467–3480, https://doi.org/10.1002/2013WR014664, 2014.

Yamazaki, D., Ikeshima, D., Tawatari, R., Yamaguchi, T., O’Loughlin, F., Neal, J. C., Sampson, C. C., Kanae, S., and Bates, P. D.: A high-accuracy map of global terrain elevations: Accurate Global Terrain Elevation map, Geophys. Res. Lett., 44, 5844–5853, https://doi.org/10.1002/2017GL072874, 2017.

Yamazaki, D., Ikeshima, D., Sosa, J., Bates, P. D., Allen, G. H., and Pavelsky, T. M.: MERIT Hydro: A High‐Resolution Global Hydrography Map Based on Latest Topography Dataset, Water Resour. Res., 55, 5053–5073, https://doi.org/10.1029/2019WR024873, 2019.
